# Absence of anti-hypocretin receptor 2 autoantibodies in post pandemrix narcolepsy cases

**DOI:** 10.1371/journal.pone.0187305

**Published:** 2017-12-08

**Authors:** Guo Luo, Ling Lin, Louis Jacob, Mélodie Bonvalet, Aditya Ambati, Giuseppe Plazzi, Fabio Pizza, Ryan Leib, Christopher M. Adams, Markku Partinen, Emmanuel Jean-Marie Mignot

**Affiliations:** 1 Stanford University Center for Sleep Sciences, Department of Psychiatry and Behavioral Sciences, Stanford University School of Medicine, Palo Alto, California, United States of America; 2 Department of Biomedical and Neuromotor Sciences, University of Bologna, Bologna, Italy; 3 Institute of the Neurological Sciences, Bologna, Italy; 4 Stanford University Mass Spectrometry, Palo Alto, California, United States of America; 5 Helsinki Sleep Clinic, Helsinki, Finland; Instituto Butantan, BRAZIL

## Abstract

**Background:**

A recent publication suggested molecular mimicry of a nucleoprotein (NP) sequence from A/Puerto Rico/8/1934 (PR8) strain, the backbone used in the construction of the reassortant strain X-179A that was used in Pandemrix^®^ vaccine, and reported on anti-hypocretin (HCRT) receptor 2 (anti-HCRTR2) autoantibodies in narcolepsy, mostly in post Pandemrix^®^ narcolepsy cases (17 of 20 sera). In this study, we re-examined this hypothesis through mass spectrometry (MS) characterization of Pandemrix^®^, and two other pandemic H1N1 (pH1N1)-2009 vaccines, Arepanrix^®^ and Focetria^®^, and analyzed anti-HCRTR2 autoantibodies in narcolepsy patients and controls using three independent strategies.

**Methods:**

MS characterization of Pandemrix^®^ (2 batches), Arepanrix^®^ (4 batches) and Focetria^®^ (1 batch) was conducted with mapping of NP 116I or 116M spectrogram. Two sets of narcolepsy cases and controls were used: 40 post Pandemrix^®^ narcolepsy (PP-N) cases and 18 age-matched post Pandemrix^®^ controls (PP-C), and 48 recent (≤6 months) early onset narcolepsy (EO-N) cases and 70 age-matched other controls (O-C). Anti-HCRTR2 autoantibodies were detected using three strategies: (1) Human embryonic kidney (HEK) 293T cells with transient expression of HCRTR2 were stained with human sera and then analyzed by flow cytometer; (2) *In vitro* translation of [^35^S]-radiolabelled HCRTR2 was incubated with human sera and immune complexes of autoantibody and [^35^S]-radiolabelled HCRTR2 were quantified using a radioligand-binding assay; (3) Optical density (OD) at 450 nm (OD450) of human serum immunoglobulin G (IgG) binding to HCRTR2 stably expressed in Chinese hamster ovary (CHO)-K1 cell line was measured using an in-cell enzyme-linked immunosorbent assay (ELISA).

**Results:**

NP 116M mutations were predominantly present in all batches of Pandemrix^®^, Arepanrix^®^ and Focetria^®^. The wild-type NP_109-123_ (ILYDKEEIRRIWRQA), a mimic to HCRTR2_34-45_ (YDDEEFLRYLWR), was not found to bind to DQ0602. Three or four subjects were found positive for anti-HCRTR2 autoantibodies using two strategies or the third one, respectively. None of the post Pandemrix^®^ narcolepsy cases (0 of 40 sera) was found positive with all three strategies.

**Conclusion:**

Anti-HCRTR2 autoantibody is not a significant biological feature of narcolepsy or of post Pandemrix^®^ autoimmune responses.

## Introduction

Although there is strong genetic evidence suggesting autoimmunity in type 1 narcolepsy [1–3], notably a 97% association with human leukocyte antigen (HLA) class II alleles DQB1*06:02 and DQA1*01:02 that together form the heterodimer DQ0602 [4], the nature of the autoimmune antigen leading to hypocretin cell death has been elusive. Renewed interest in this area came from the observation of increased incidence of type 1 narcolepsy following the 2009–2010 winter vaccination campaign against the pandemic H1N1 (pH1N1) “swine” flu (pH1N1-2009) that emerged in 2009 [5]. This effect was mostly obvious in Scandinavia, where vaccine coverage with a specific adjuvant system 03 (AS03) (composed of α-tocopherol, squalene and polysorbate 80) adjuvanted vaccine Pandemrix^®^ from GlaxoSmithKline (GSK) was high (~50%), and rapid onset narcolepsy was frequently observed in children within a few months following vaccination [5–10]. In Finland, where incidence of narcolepsy was best estimated and most consistent with known population prevalence (0.02%) [11], risk of Pandemrix^®^ induced narcolepsy increased ~10 fold, although absolute risk of developing narcolepsy still remained low (less than 10 for 100,000 vaccinees versus (vs) 0.7 for 100,000 baseline incidences) [12]. Others also found that the onset of narcolepsy was frequently seasonal [13], occurring in spring or summer, and following winter upper-airway infections such as *Streptococcus Pyogenes* [14, 15]. Observation of seasonality of onset was more obvious in young children where narcolepsy onset is very abrupt and can more easily be timed to the exact month. All together, these data strongly suggest that narcolepsy is an autoimmune disease triggered by upper airway infections, notably influenza and maybe *Streptococcus Pyogenes*, with stronger susceptibility to pH1N1-2009.

Additional surprising or inconsistent observations were made thereafter. First, whereas in China, a clear increase in childhood narcolepsy cases was observed in ecological studies of two sleep centers during the winter of 2009–2010 [13, 16, 17], a weaker effect was observed in Germany [18] and in the United States [19, 20]. Second, increased onset of narcolepsy independent of vaccination was not observed in Scandinavia [21], although this could be due to herd immunity protection against pH1N1 through the high coverage vaccination campaign. Third, Pandemrix^®^ vaccination-induced narcolepsy seemed to have occurred more frequently in some areas of Sweden, notably in the south of the country, possibly reflecting differential timing of the infection vs the vaccination campaign, or possible differences between vaccine batches [22, 23]. Fourth, other pH1N1 vaccines were not associated with increased risk of developing narcolepsy, notably in the United States, where only sporadic cases have been reported after flu vaccinations. As only unadjuvanted or live attenuated vaccines were used during the 2009–2010 vaccination campaign in the United States, this suggests that adjuvant AS03 played a role in Pandemrix^®^ vaccination-induced narcolepsy. Surprisingly, however, two other adjuvanted pH1N1 vaccines used in countries other than Scandinavia had little or no effects: one is MF59C.1 (containing squalene, polysorbate 80 and sorbitan trioleate) adjuvanted Focetria^®^ from Novartis used in Europe (mostly in Italy) [24], the other is Arepanrix^®^ used in Canada and South America [25], another AS03 adjuvanted vaccine from GSK that is almost identical to Pandemrix^®^ except for the method of antigen isolation.

Risk of narcolepsy remained low even with Pandemrix^®^ vaccination in 2009–2010 and DQ0602 positive. Indeed, narcolepsy occurred in vaccinated individuals with an incidence of less than 1/3,000 persons after Pandemrix^®^ even in DQ0602 positive individuals. The reason for that narcolepsy incidence rate following pH1N1 vaccination varied across vaccines is likely multifactorial, and may involve vaccine composition differences (adjuvant and antigen isolation protocol), prior immune history and genetics of targeted populations. It is notable that some targeted subgroups were prioritized in each vaccination campaign (children vs adults, high risk groups) and that timing of vaccination in relation to the unfolding pandemic flu occurred in close temporal successions, usually within a few weeks in many countries. Nonetheless, one of the most reliable findings may be that Pandemrix^®^ had strong effects in most European countries [26–31], and that Arepanrix^®^, a closely related vaccine had less or no effects in Quebec and Canada, suggesting that differences in vaccine composition are involved, as suggested by Ahmed *et al*. [32].

Following on these observations, several authors went on to compare the composition of known pH1N1 monovalent vaccines, with primary focus on Pandemrix^®^ and Arepanrix^®^, the two most closely related vaccines with differential risks [33]. The creation of flu vaccine is a long process that involves growing genetically engineered flu strains in *Gallus gallus* (chicken) eggs, and purifying vaccine particles and antigens for vaccine preparation, with primary focus on Hemagglutinin (HA) and Neuraminidase (NA), two surface proteins that are strongly involved in lymphocyte B cell antibody (Ab) responses to the flu [34–37]. The pH1N1-like vaccine strains X-179A and X-181 were created in New York Medical College (NYMC) for the 2009–2010 swine flu campaign, using an old H1N1-1918-like strain, A/Puerto Rico/8/1934 (PR8), as a backbone, and reassorted with A/California/07/2009 [38]. In both X-179A and X-181 strains, HA, NA and polymerase (basic) protein 1 (PB1) were derived from A /California/07/2009, while other proteins were PR8-derived [39, 40]. NYMC X-181, characterized by a mutation of HA 146N to 146D and a few other differences (including HA titer, yield of purified viral protein) [41], was created in late 2009 and selected because it produced higher reassortant HA titers than X-179A. It was used toward the end of the season (2009) in some cases [38–40]. Both Pandemrix^®^ and Arepanrix^®^ were produced by GSK using X-179A, while Focetria^®^ was created by Novartis using X-181, with their own patented processes. Jacob *et al*. analyzed the compositions of Pandemrix^®^ (2009 batches) and Arepanrix^®^ (a 2010 batch) using MS and showed results obtained with five main viral proteins: HA1 and HA2 subunits of HA, NA, NP, matrix protein 1 (M1), and non-viral proteins from chicken growth matrix [33]. Overall, these vaccines were remarkably similar. Pandemrix^®^ contained slightly more NP and NA, while Arepanrix^®^ displayed a larger diversity of viral and chicken proteins, with the exception of five chicken proteins that were relatively more abundant in Pandemrix^®^ [33]. Interestingly, HA1 146N (HA 146N) (amino acid residue 129N in the mature protein) was found to be mutated to 146D in most of the HA antigens of Arepanrix^®^ [33], a surprising finding as both Pandemrix^®^ (2009 and 2010 batches) and Arepanrix^®^ (a 2010 batch) were manufactured using X-179A. This last finding suggested that the HA 146N to 146D mutation emerged during Arepanrix^®^ manufacturing prior to 2010, and raised the possibility that this mutation could have played a role in differential vaccine susceptibility. The main limitation of this study was the use of a 2010 batch of Arepanrix^®^, which was produced one year later than the ones used during the 2009–2010 vaccination campaign [33].

In parallel with Jacob *et al*. findings [33], Vaarala *et al*. studied post Pandemrix^®^ patient and control antibody reactivity against Pandemrix^®^ and Arepanrix^®^, and found that Arepanrix^®^ reacted poorly with antibodies of post Pandemrix^®^ vaccinated children, suggesting antigenic differences in antibody determinants [42]. Increased antibody levels to HA and NP, particularly to structurally altered viral NP, were also seen in post Pandemrix^®^ narcoleptic children [42]. The authors went on to hypothesize that the higher amount of denatured NP in Pandemrix^®^ during the purification process of viral proteins may explain increased narcolepsy susceptibility with Pandemrix^®^ vaccination.

Following on these observations, Ahmed *et al*. identified a sequence of NP_111-121_ (YDKEEIRRIWR) with NP 116I (underlined) in X-179A with significant homology to a sequence of the first extracellular domain of HCRTR2 (HCRTR2_34-45_, YDDEEFLRYLWR), whereas a mutation of NP 116I to 116M in NP_111-121_ (YDKEEMRRIWR, 116M was underlined) was found in Focetria^®^, which in any case contained only very limited amount of NP because of its specific subunit compositions [43]. The authors also found anti-HCRTR2 autoantibodies were detectable in post Pandemrix^®^ narcolepsy patients (17 of 20 sera), but not in subjects (0 of 12 sera) after Focetria^®^ vaccination in 2009.

This article raised two interesting points: (1) the possibility of amino acid residue mutations, such as NP 116I to 116M in some vaccine strains, may influence vaccine response and mimicry; (2) a possible role of anti-HCRTR2 autoantibodies in the pathophysiology of narcolepsy [43]. In this study, we further evaluated these hypotheses by (1) examining the prevalence of NP 116I and 116M in Pandemrix^®^, Arepanrix^®^ and Focetria^®^ using MS characterization and (2) testing for the presence of anti-HCRTR2 autoantibodies using three independent strategies in post Pandemrix^®^ narcolepsy (PP-N) patients, recent early onset narcolepsy (EO-N) patients, post Pandemrix^®^ controls (PP-C), and other controls (O-C).

## Materials and methods

### Ethics statement

This study was reviewed and approved by the Stanford University Institutional Review Board (Protocol # 14325, Registration # 5136). Informed consent was obtained from each participant.

### Participants

Two groups of matched narcolepsy patients and controls were created. All patients meet international classification of sleep disorders 3 (ICSD3) criteria (http://www.aasmnet.org/store/product.aspx?pid=849) for type 1 narcolepsy and were DQ0602 positive with one exception, a patient known to have low cerebrospinal fluid (CSF) hypocretin-1 levels (S1 Table). Narcolepsy patients included 40 post Pandemrix^®^ cases (mean age ± standard error of the mean (SEM): 14.9 ± 1.26, 30% male) and 48 recent early onset cases (mean age ± SEM: 12.95 ± 1.43, 58% male). Controls included 18 post Pandemrix^®^ subjects (mean age ± SEM: 13.17 ± 0.97, 39% male) and 70 other control subjects (mean age ± SEM: 18.85 ± 1.31, 46% male) (S1 Table).

### Vaccines

Antigen samples of Pandemrix^®^ (2 batches, Lot# AFLSA208A and AFLSFDA280), Arepanrix^®^ (4 batches, lot# AFLPA328AA, AFLPA359AA, AFLPA373BA, and SF1B0454CL), and Focetria^®^ (1 batch, Lot# E53P22B) were provided by GSK or vaccine centers around the world. The two Pandemrix^®^ lots and four Arepanrix^®^ lots (lot# SF1B0454CL was analyzed by Jacob *et al*. [33]) studied here have been used during the 2009–2010 vaccination campaign in Europe and Canada, respectively. Vaccine compositions were summarized by European medicines agency or GSK [44, 45].

### Mass spectrometry (MS)

Detailed protocols have been published previously [33]. Briefly, MS was performed on Trypsin/Lys-C mix (Cat# V5073, Promega) and chymotrypsin (Cat# V1062, Promega) digests of Pandemrix^®^, Arepanrix^®^ and Focetria^®^ (6.5 μg) samples. Each vaccine was first precipitated using 4× volume of high-performance liquid chromatography (HPLC) grade acetone at -80°C, followed by overnight precipitation. Centrifugation at 4°C, 12000× *g* for 15 minutes was performed, supernatant removed and protein pellets dried. Pellets were digested (1:25 of protease to protein ratio at 37°C for 4–18 hours), then quenched by the addition of 10% formic acid. Resulting peptides were cleaned on a micro spin column (Cat# NC9270379, Nest group Inc.) and dried by speed-vacuum. Samples were then reconstituted in 20 μL 2% acetonitrile, 0.1% formic acid, and 97.9% water, and 3 μL of sample was injected onto a self-packed 15 cm C18 reverse phase column (Easy-nLC II, ThermoFisher Scientific) where a linear gradient from 3–40% mobile phase B was used over 2 hours to elute peptides into the mass spectrometer. Sample ionization was done using a spray voltage of 1.7 kilovoltage (kV). The mass spectrometer was an LTQ Orbitrap Velos or Orbitrap Fusion (ThermoFisher Scientific) set to acquire in a data dependent acquisition (DDA) mode, in which the top 12 most intense multiply charged precursor ions were selected for fragmentation by the ion trap. The target ion values (AGC settings) were 7.5×10^5^ and 2.5×10^4^ for the orbitrap and the ion trap, respectively.

### MS analysis of HA and NP proteins

Raw MS data of these vaccines (S1 and S2 Raw Data) were analyzed using a combination of Byonic^®^ and Preview^®^ software (version1.4, Protein Metrics). Representative MS spectra were exported from Byonic^®^. Data analysis with Preview^®^ was completed for each sample using a concatenated FASTA file containing the canonical proteomes for five influenza viral strains (A/California/07/2009, NYMC X-181A (identical to NYMC X-181), NYMC X-179, NYMC X-179A, and A/Puerto Rico/8/1934). NCBI accession numbers of X-179A viral strains we used for peptide mapping were ADE2909 (used in Ahmed *et al*. [43] study) (Table 1) and AIE5269, which was our more recently released (May 2014) and contained an NP 116M (Table 1). These two X-179A sequences contained identical alleles of HA 146N. The X-181 sequence we used was AFM7284 (June 2012) (Table 1), which contained HA 146D and NP 116M [33, 43]. All statistical analyses were performed on raw data and were undertaken using Cochran-Mantel-Haenszel chi square tests using RStudio 3.1.0, using mantelhaen.test and cmh.test functions [46].

**Table 1:** NCBI accession numbers and major polymorphisms found in pH1N1 influenza A viral strains used in our MS data analysis.

Strains	Accession numbers	Publication date	Submission	HA			NP			
				Accession numbers	%identity	146	Accession numbers	%identity	116	130
A/California/07/2009*	AFM728	20 June 2012	JCVI	AFM72832.1	100%	N	AFM72836.1	100%	I	T
A/Puerto Rico/8/1934	NP_04098	12 June 2000	NCB	NP_040980.1	82.3%	T	NP_040982.1	91.6%	I	T
A/reassortant/NYMC X-179 (A/California/07/2009 x NYMC X-157^‡^)	ADE2908	30 March 2010	NYMC	ADE29085.1	99.6%	N	ADE29086.1	91.6%	I	T
A/reassortant/NYMC X-179 (A/California/07/2009 x NYMC X-157)	AIE525	23 May 2014	JCVI	AIE52549.1	99.8%	N	AIE52553.1	91.6%	I	T
A/reassortant/NYMC X-179A (A/California/07/2009 x NYMC X-157)	ADE2909	30 March 2010	NYMC	ADE29095.1	99.8%	N	ADE29096.1	91.4%	I	A
A/reassortant/NYMC X-179A (A/California/07/2009 x NYMC X-157)	AIE5269	23 May 2014	JCVI	AIE52692	99.8%	N	AIE52696.1	91.4%	M	T
A/reassortant/NYMC X-181(A/California/07/2009 x NYMC X-157)	AFM7284	20 June 2012	JCVI	AFM72842.1	99.6%	D	AFM72846.1	91.4%	M	T
A/reassortant/NYMC X-181A (A/California/07/2009 x NYMC X-157)	AFM7285	20 June 2012	JCVI	AFM72852.1	99.6%	D	AFM72856.1	91.4%	M	T

*was used as the reference strain for the establishment of percentage of identity.

ADE2908 and AFM7284 were identical to AIE525 and AFM7285, respectively. AIE5269 and ADE2909 displayed six amino acid residue differences (two in NP and four in nonstructural protein 1 (NS1)).

^‡^See Fulvini *et al*. publication [38].

### HA and NP peptides binding to DQ0602 monomers

Complementary deoxyribonucleic acid (cDNA) templates of HLA-DQA1*01:02 and DQB1*06:02 were obtained from the Emory University NIH core tetramer facility (http://tetramer.yerkes.emory.edu/support/faq). Soluble DQ0602 monomers were expressed in “high five” cell line (a gift from K. Christopher Garcia lab, Stanford University) and purified using fast protein liquid chromatography (FPLC). The CLIP peptide, which derives from the major histocompatibility complex (MHC) class II-associated invariant chain (li), was removed by cleavage with thrombin (Cat# 69671, EMD Millipore). CLIP plays a critical role in the assembly of MHC, especially for antigen processing by stabilizing peptide-free DQ0602 complex [47–49]. Another nonclassical MHC class II heterodimer molecule, HLA-DM, which regulates and catalyzes antigenic peptide loading onto DQ0602 [50, 51], was also expressed in “high five” cell line and purified. Peptides were ordered at GenScript company with >90% purity and dissolved in dimethyl sulfoxide (DMSO) at stock concentration of 10 mM.

Peptide competing binding assays were conducted by incubation of 25 nM DQ0602, 100 nM HLA-DM, 1 μM biotin-conjugated Epstein-Barr virus (EBV) epitope (Bio-EBV, Bio-GGGRALLARSHVERTTDE), with 40 μM of the competitor peptide in reaction buffer (100 mM acetate, pH = 4.6, 150 mM NaCl, 1% BSA, 0.5% Nonidet P-40 (IGEPAL CA-630, Sigma), 0.1% NaN_3_) in duplicate for 3 days at 37°C. The reaction was quenched by adding two volumes of neutralization buffer (100 mM Tris-HCl, pH = 8.6, 150 mM NaCl, 1% BSA, 0.5 Nonidet P-40 (IGEPAL CA-630, Sigma), 0.1% NaN_3_). Monoclonal anti-DQ (SPV-L3) antibodies (Cat# BNUM0200-50, Biotium) (1:400 dilution in 100 mM carbonate-bicarbonate buffer, pH = 9.5) were coated onto a high binding 96-well plate (REF# 9018, Corning), and incubated with neutralized reaction for 1–2 hour at room temperature (RT). After washing five times with 300 μl/well of wash buffer (phosphate-buffered saline (PBS), 0.05% Tween-20, pH = 7.4), 100 μl/well of Europium (Eu)-labelled streptavidin (Cat# 1244–360, PerkinElmer) (1:1000 dilution in PBS, 1% BSA, pH = 7.4) was added and incubated for 1 hour at RT. After washing 5 times again with 300 μl/well of wash buffer, DELFIA^®^ time-resolved fluorescence (TRF) intensity was detected using a Tecan Infinite^®^ M1000 after adding 100 μl/well of enhancement solution (Cat# 1244–105, PerkinElmer). Non-specific binding was removed through extensive wash with wash buffer. Competitor peptide with Eu TRF intensity that was lower than 25% of Bio-EBV epitope alone was considered strong binder, while peptide with 25–50% was weak binder.

### HCRTR2 constructs

For HCRTR2 transcription and translation *in vitro*, plasmid *pCMV3-HA-HCRTR2* was purchased from Sino Biological Inc. (Cat# HG10844-NY-HCRTR2). For immunofluorescence observation and flow cytometry analysis, construct *pcDNA3*.*1-HCRTR2-GFP* was generated as follows: coding sequence (CDS) of *HCRTR2* was amplified with high fidelity Platinum^®^ *Taq* DNA polymerase (Cat# 11304011, ThermoFisher Scientific) (forward primer, 5’-GTGATGTCCGGCACCAAAT-3’, reverse primer, 5’-CCCAGTTTTGAAGTGGTCCTG-3’) using plasmid *pCMV3-HA-HCRTR2* as template and then fused to N terminus of the *green fluorescent protein (GFP)* gene using a CT-GFP fusion TOPO^®^ TA cloning kit (Cat# K482001, ThermoFisher Scientific). The insertion was confirmed by deoxyribonucleic acid (DNA) sequencing.

### Anti-HCRTR2 autoantibody detection with flow cytometry

HEK293T cells (purchased from ATCC, https://www.atcc.org/Products/All/CRL-3216.aspx) [52, 53] were cultured in Dulbecco’s modified Eagle’s medium (DMEM) (ATCC^®^ 30–2002, ATCC) supplemented with 10% fetal bovine serum (FBS) (Cat# 26140079, ThermoFisher Scientific) and 1% penicillin-streptomycin (P/S) (Cat# 10378016, ThermoFisher Scientific) in 75-centimeter (cm)^2^ tissue culture flask (REF# 658175, CELLSTAR) at 37°C, 5% CO_2_ and transfected with *pcDNA3*.*1-HCRTR2-GFP* using the Lipofectamine^®^ 3000 reagent (Cat# L3000015, ThermoFisher Scientific) in Opti-MEM^®^ I reduced serum medium (REF# 31985070, ThermoFisher Scientific). After 24 hours, cells were harvested and stained in 100 μL staining buffer (Cat# 420201, Biolegend) with human serum at 1:20 dilution or mouse monoclonal positive anti-HCRTR2 antibody (Cat# WH0003062M1-100UG, Sigma-Aldrich) at different dilution ratios for 1 hour at 4°C. All serum samples were clarified by centrifugation for 10 minutes at 8,000× *g*. After washing twice with staining buffer, cells were stained with Alexa Fluor^®^ 555 (AF555)-conjugated goat anti-human IgG (H+L) antibody (Cat# A-21433, ThermoFisher Scientific) or goat anti-mouse IgG (H+L) antibody (Cat# A-21422, ThermoFisher Scientific) at 1:100 dilution. After washing twice with staining buffer, propidium iodide (PI, Sigma-Aldrich) was added to each sample to separate live cells from dead cells. Samples were then run on a BD flow cytometer LSRII. Data analyses were performed with FlowJo software (version 10.0.8, FlowJo LLC). The sensitivity of the AF555 channel fluorescence intensity was tested using positive control antibodies (Cat# WH0003062M1-100UG, Sigma-Aldrich) at different dilution ratios (S1 Fig, S2 Table).

Methods for quantitation analysis have been described previously [54, 55] and were modified as follows: for each sample, mean fluorescence intensities (MFI) of AF555 channel (MFI^AF555^) within live GFP positive HEK293T cells (HEK293T^GFP+^) and GFP negative HEK293T cells (HEK293T^GFP-^) were calculated, then ΔMFI^AF555^ was determined by subtracting MFI^AF555^ of HEK293T^GFP-^ from MFI^AF555^ of HEK293T^GFP+^. A staining control with positive antibody at 1:100 dilution was included in each staining experiment. The MFI^AF555^ index was calculated as follows: 100× (ΔMFI^AF555^ of each sample)/(ΔMFI^AF555^ of positive antibody at 1:100 dilution). Cut-off value for a positive MFI^AF555^ index is determined as the mean of MFI^AF555^ + 3× standard deviation (SD) of control subjects [56].

### Anti-HCRTR2 autoantibody detection using [^35^S]-radiolabelled HCRTR2

For detailed methods, see Tanaka *et al*. [56]. Briefly, [^35^S]-radiolabelled HCRTR2 was synthesized using the TNT^®^ quick coupled transcription/translation system (Cat# L1170, Promega), with *pCMV3-HA-HCRTR2* as the template, and [^35^S]-methionine (Cat# NEG009L005MC, PerkinElmer) as the labeling radioisotope, according to the manufacturer’s instructions. Free [^35^S]-methionine was removed using an illustra^®^ NICK^®^ column (Cat# 17-0855-01, GE Healthcare). Elution fractions were loaded into mini-PROTEAN^®^ TGX^®^ precast gel (Cat# 4561093, Bio-Rad Laboratories, Inc.) and sodium dodecyl sulfate polyacrylamide gel electrophoresis (SDS-PAGE) analysis was carried out. The expression of [^35^S]-radiolabelled HCRTR2 was confirmed with radioactive exposure of x-ray film (Product code# 28906838, GE Healthcare) (S2 Fig). Fractions that contained [^35^S]-radiolabelled HCRTR2 were combined and adjusted to 20,000 counts per minute (cpm) per 20 μL concentration with reaction buffer (50 mM Tris-HCl, 150 mM NaCl, 0.1% BSA, 0.1% Tween-20, and 0.1% NaN_3_, pH = 7.4). Positive anti-HCRTR2 antibody (Cat# ab65093, Abcam) reaction sensitivity was tested (S3 Table). 12 random sera, including 6 patients and 6 controls, with five dilution ratios from 1:50 to 1:800 in reaction buffer were also tested (S3 Table). All sera were diluted at 1:50 for quantitation as follows: 1 μL of serum was incubated with 20,000 cpm of [^35^S]-radiolabeled HCRTR2 in 50 μL reaction mixture (including 29 μL reaction buffer) overnight at 4°C. The reaction mixture was transferred to a Multiscreen^®^ filter plate (Cat# MSHVN4B10, EMD Millipore). 10 μL of protein G Sepharose^®^ (Cat# 17-0618-01, GE Healthcare) was added to each well. The plate was incubated for 1 hour at RT, and stringently washed 10 times with 300 μL/well of wash buffer (50 mM Tris-HCl, 150 mM NaCl, and 1% Tween-20, pH = 7.4) using a vacuum manifold (EMD Millipore). The plate was dried, and MicroScint^®^-O cocktail (Product# 6013511, PerkinElmer) was added to each well. The plate was read by a TopCount NXT^®^ apparatus (PerkinElmer). Each serum or positive antibody (Cat# ab65093, Abcam) was duplicated. Anti-HCRTR2 autoantibody index was calculated as follows: (cpm of serum)/(mean cpm of reaction buffer). The cut-off value for a positive sample is determined as the mean + 3× SD of control samples [56].

### Anti-HCRTR2 autoantibody detection using in-cell ELISA

In-cell ELISA colorimetric assay was performed according to the manufacturer’s instructions (Cat# 62200, ThermoFisher Scientific). CHO-HCRTR2 cell line was made in a CHO-K1 host cell line (purchased from ATCC, https://www.atcc.org/products/all/CCL-61.aspx) [57, 58] and cultured in Kaighn’s modification of Ham’s F-12 (F-12K) medium (ATCC^®^ 30–2004, ATCC) supplemented with 10% FBS (Cat# 26140079, ThermoFisher Scientific) and 1% P/S (Cat# 10378016, ThermoFisher Scientific) at 37°C, 5% CO_2_. Transgenic HCRTR2 expression was detected by flow cytometry (S3 Fig) and western blotting (S4 Fig). Cell number sensitivity, from 625 to 100,000 live CHO-HCRTR2 cells per well, was tested with a series of positive antibody (Cat# ab65093, Abcam) concentrations (S4 and S5 Tables), and 10,000 live CHO-HCRTR2 cells per well were used in all the other in-cell ELISA assays. In order to verify performance of the in-cell ELISA assay, two additional positive antibodies (Cat# 07505–20, United States Biological, Cat# AP55124SU-N, Acris) present in rabbit sera were also tested with titration (S6 Table). Briefly, 10,000 live CHO-HCRTR2 cells/well were plated in a black collagen I-coated 96-well plate (REF# 152035, ThermoFisher Scientific), and cultured overnight at 37°C, 5% CO_2_. Cells were fixed with 4% formaldehyde (Cat# 28906, ThermoFisher Scientific) for 15 minutes at 4°C in the dark, followed by blocking overnight at 4°C with 200 μL/well of blocking buffer (included in kit). 30 concentrations of positive antibodies (Cat# ab65093, Abcam) in duplicate were made to generate standard curve using 4-parameter logistic fit by SoftMax^®^ Pro software (version 5.4, Molecular Devices) (S7 and S8 Tables). In order to make all sera (2 samples running out) fall within a standard range, sera were diluted at different ratios in 1× tris-buffered saline (TBS) buffer (included in kit) and added to plates in duplicate for incubation overnight at 4°C (S7 and S8 Tables). After washing the plates three times with 200 μL/well of 1× wash buffer (prepared according to the manufacturer’s instruction), 100 μL/well of diluted horseradish peroxidase (HRP) conjugate was added and incubated for 30 minutes at RT. The plates were then washed three times with 200 μL/well of 1× wash buffer, and 100 μL/well of 3,3’,5,5’-tetramethylbenzidine (TMB) substrate (included in kit) was added. The solution was incubated in the dark at RT and the reaction was stopped within 15 minutes by adding 100 μL/well of TMB stop solution (included in kit). The plate signal was read immediately using a SpectraMax^®^ M2 plate reader (Molecular Devices) at 450 nm. The original OD450 of each serum was analyzed using the SoftMax^®^ Pro software (version 5.4, Molecular Devices) and corrected to that at 1:800 dilution (S7 and S8 Tables). The OD450 shown in related graphs or figures is determined by subtracting OD450 of 1× TBS buffer in the same plate from corrected OD450 of each serum. The cut-off value for a positive sample is determined as the mean + 3× SD of control samples [56].

### Western blotting

A standard protocol for western blotting was used (http://www.bio-rad.com/webroot/web/pdf/lsr/literature/Bulletin_6376.pdf). Briefly, 8.163 millions of live CHO-K1 or CHO-HCRTR2 cells were collected and lysed with RIPA buffer (Cat# 8900, ThermoFisher Scientific) supplemented with complete protease inhibitor (REF# 11873580001, Roche Diagnostic GmbH). Debris were removed by centrifugation at 12,000 rpm for 15 minutes. 15 μL of whole cell lysates mixed with 5 μL of 4× Laemmli buffer (Cat# 1610747, Bio-Rad Laboratories, Inc.) were loaded into mini-PROTEAN^®^ TGX^®^ precast gel (Cat# 4561093, Bio-Rad Laboratories, Inc.). After transferring proteins from the gel to an Immobilon^®^-P membrane (Cat# IPVH00010, EMD Millipore) and blocking the membrane with a blocking buffer (Cat# 37543, ThermoFisher Scientific), the primary anti-HCRTR2 antibody (Cat# WH0003062M1-100UG, Sigma-Aldrich) (1:500 dilution) was incubated for 2 hours at RT, followed by incubation with a secondary, peroxidase-conjugated, anti-mouse IgG (H+L) antibody (Code# 715-035-150, Jackson ImmnuoResearch Laboratories, Inc.) (1:5000 dilution). Non-specific binding was removed through washing 3 times for 10 minutes (PBS, 0.05% Tween-20). Chemiluminescent substrate (Prod# 32106, ThermoFisher Scientific) was applied to the blot following the manufacturer’s instructions. Chemiluminescence film (Product code# 28906838, GE Healthcare) was developed using a tablet film processor (Item# SRX-101A, Konica).

### Cell-base assay (CBA) for anti-HCRTR2 autoantibody detection under microscope

HEK293T cells (purchased from ATCC, https://www.atcc.org/Products/All/CRL-3216.aspx) [52, 53] were cultured in DMEM (ATCC^®^ 30–2002, ATCC) supplemented with 10% FBS (Cat# 26140079, ThermoFisher Scientific) and 1% P/S (Cat# 10378016, ThermoFisher Scientific) at 37°C, 5% CO_2_ in a 12-well plate (REF# 353043, Corning) with one glass coverslip (REF# 354085, Corning) per well. These cells were then transfected with *pcDNA3*.*1-HCRTR2-GFP* using the Lipofectamine^®^ 3000 reagent (Cat# L3000015, ThermoFisher Scientific) in Opti-MEM^®^ I reduced serum medium (REF# 31985070, ThermoFisher Scientific). After 24 hours, cells were washed with PBS and fixed with 4% formaldehyde (Cat# 28906, ThermoFisher Scientific) for 15 minutes at 4°C in the dark, followed by blocking for 1 hour at RT in the dark in saturation buffer (PBS, 0.2% gelatin (Cat# G7041-100G, Sigma-Aldrich)). Cells were stained with human sera at 1:20 dilution or with positive control antibodies (Cat# ab65093, Abcam) at 1:100 dilution for 2 hours at RT in the dark. All sera were clarified by centrifugation for 10 minutes at 8,000× *g*. Cells were then stained for 1 hour at RT in the dark with Alexa Fluor^®^ 555-conjugated goat anti-human IgG (H+L) antibody (Cat# A-21433, ThermoFisher Scientific) or donkey anti-goat IgG (H+L) antibody (Cat# A-21432, ThermoFisher Scientific) at 1:1000 dilution, respectively, and also incubated with 4′,6-Diamidino-2-Phenylindole dihydrochloride (DAPI). Images were taken by EVOS^®^ FL imaging system (Cat# AMF4300, ThermoFisher Scientific). All antibodies were diluted in saturation buffer and non-specific binding was removed through extensive wash 3 times with PBS.

### Statistical analyses

For MS analysis, categorical values were expressed as percentages, linear values as mean ± SD or standard error. Data were analyzed using SAS software (SAS Institute Inc.) with two-sided *t*-tests. P-value <0.05 was considered statistically significant.

For anti-HCRTR2 autoantibody analysis and peptide binding assay, values were expressed as mean ± SD, and statistical comparisons were calculated using two-tailed *t*-tests in Microsoft Excel. Data were plotted using GraphPad PRISM 5 (GraphPad Software, Inc.). P-value <0.05 was considered statistically significant.

## Results

### NP 116M was predominantly present in all vaccines tested

**Fig 1:**
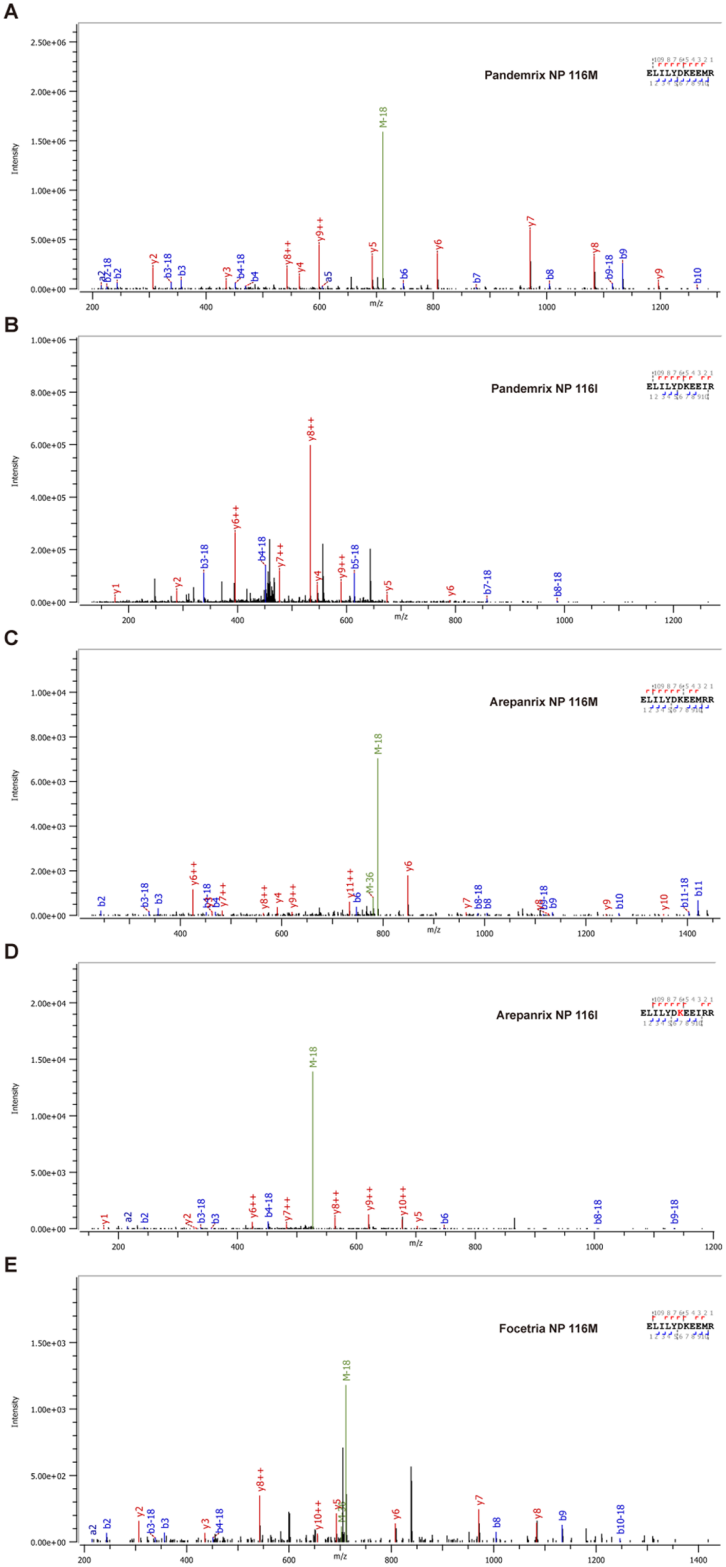
Representative MS spectra of NP peptides around NP 116I or 116M as observed in Pandemrix^®^ (A and B), Arepanrix^®^ (C and D), and Focetria^®^ (E). NP 116M was predominantly found in all the three vaccines. Due to low amount of NP in Focetria^®^, only NP 116M was found. Series a/b and y of peptides were numbered from N-terminal and C-terminal ends, respectively. Associated series number denoted size of fragments in amino acid residue number, from 1 to 10. NP 116I to 116M mutation corresponded to an 18Da shift.

Spectrograms were mapped on a library of peptides generated from the trypsin and chemotrypsin digests of X-179A and X-181 with accession numbers ADE2909 (March 2010), AIE5269 (May 2014), and AFM7284 (June 2012) (Fig 1, Table 1, S5 Fig, S9–S18 Tables). Frequencies of NP 116I and 116M, HA 146N and 146D for Pandemrix^®^, Arepanrix^®^ and Focetria^®^ were shown in Table 2, with the caveat that much lower NP coverage was obtained with Focetria^®^ (consistent with the subunit nature of this vaccine). We found that the occurrences of mutation vs wild type in Pandemrix^®^, Arepanrix^®^, and Focetria^®^ were 47 vs 2, 76 vs 3, and 5 vs 0, respectively, indicating that the NP 116M mutation was predominantly present in all the three vaccines (Fig 1, Table 2).

**Table 2:** NP 116I to 116M and HA 146N to 146D amino acid residue mutations in Pandemrix^®^, Arepanrix^®^ and Focetria^®^.

Amino acid residue in pH1N1-2009^a^	Peptide region^b^	Nucleotide mutation	DQ0602 binding^c^	Pandemrix^®^^d^	Arepanrix^®^^d^	Focetria^®^^d^
NP 116I	RELILYDKEEIRRIWRQANNG	A > G	NB > NB	95.9% (47, 49)	96.2% (76, 79)	100% (5, 5)
HA 146N	KTSSWPNHDSNKGVTAACPHA	A > G	WB > WB	6.1% (2, 33)	64.9% (24, 37)	11.8% (2, 17)

^a^The identity of each wild-type amino acid residue at the same position in the pH1N1-2009 strain (A/California/07/2009) was given.

^b^The peptide region corresponding to each amino acid residue around each wild-type amino acid residue was shown. Each wild-type amino acid residue to be mutated was underlined.

^c^Binding affinity changes from wild type to mutation were predicted by NetMHCII 2.2 server (http://www.cbs.dtu.dk/services/NetMHCII/) [59, 60]. NB, no binding; WB, weaker binding.

^d^The proportion of each mutated amino acid residue was shown with occurrences of mutation (former) and total (latter) between brackets.

Wild-type HA 146N was predominantly found in both Pandemrix^®^ (2 mutations vs 31 wild types) and Focetria^®^ (2 mutations vs 15 wild types) (Table 2), while HA 14D mutation was predominantly found in Arepanrix^®^ (24 mutations vs 13 wild types) (Table 2, S5 Fig).

### NP_109-123_ containing wild-type 116I did not bind to DQ0602

**Fig 2:**
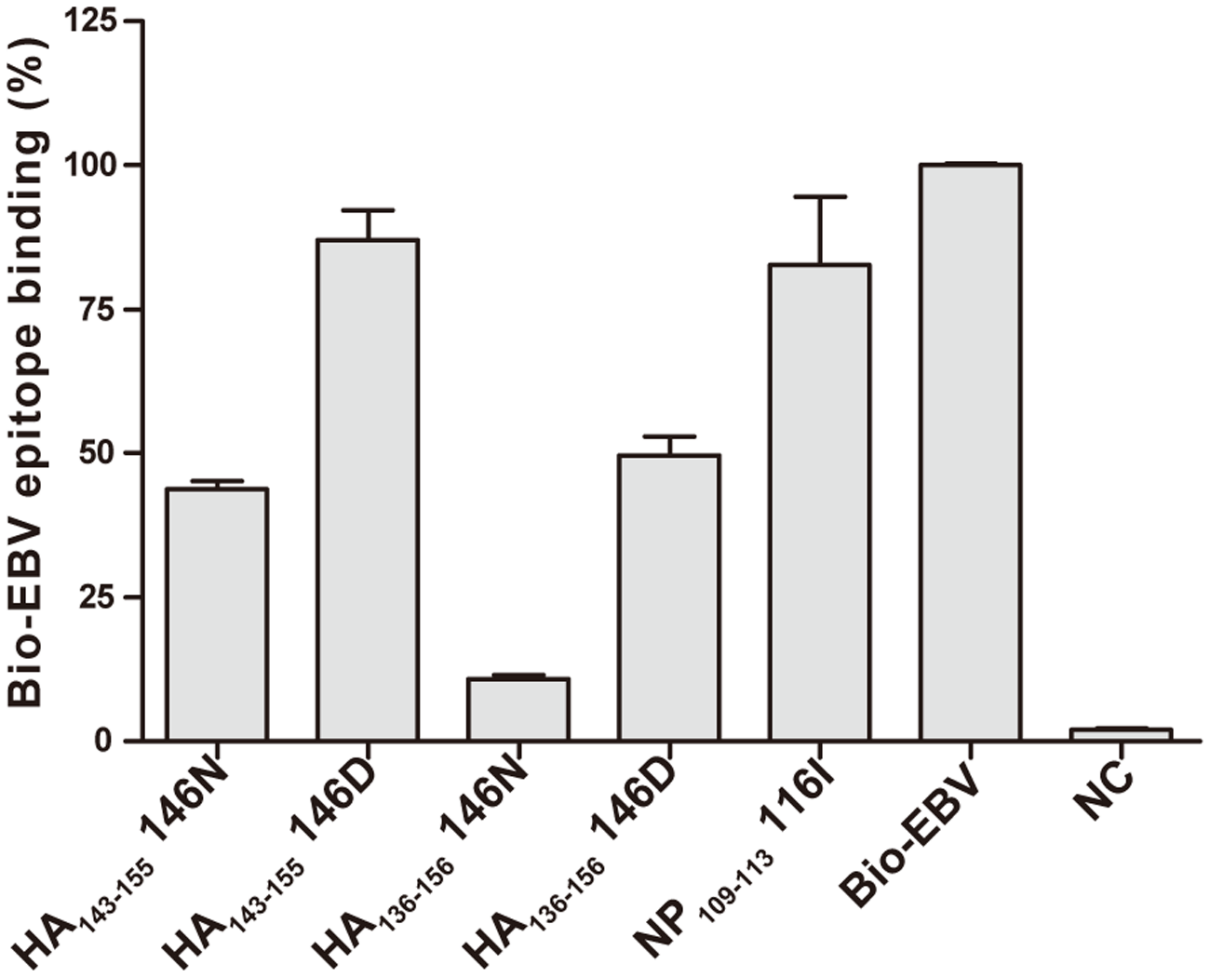
NP and HA peptides binding to DQ0602. Synthetic 15-mer peptide NP_109-123_ containing 116I, 13-mer HA_143-155_ containing 146N or 146D, and 21-mer HA_136-156_ containing 146N or 146D competed with Bio-EBV (Bio-GGGRALLARSHVERTTDE) epitope to bind to DQ0602. Time-resolved fluorescence (TRF) intensity was measured with Europium-labelled streptavidin. Stronger competitor gave lower percentage of Bio-EBV epitope binding. Peptides with <25%, 25–50% and >75% of Bio-EBV epitope binding were considered strong binder, weak binder and no binding, respectively. DQ0602 together with HLA-DM without any peptide was shown as negative control (NC).

To investigate binding affinity to DQ0602, peptide competing binding assay was performed with synthetic wild-type NP_109-123_ (ILYDKEEIRRIWRQA) peptide (116I was underlined) and HA peptides containing wild-type (146N) or mutated (146D) HA alleles each with 13-mer (HA_143-151_, HDSN/DKGVTAACPH) and 21-mer (HA_136-156_, KTSSWPNHDSN/DKGVTAACPHA) (146N/D was underlined). The results showed that wild-type NP_109-123_ didn’t bind to DQ0602 (mean of Bio-EBV epitope binding ± SD, 82.74% ± 11.71%) (Fig 2, S19 Table), and that wild-type HA peptide bound stronger than mutated HA with either 13-mer or 21-mer peptide and longer peptide contributing higher binding affinity (mean of Bio-EBV epitope binding ± SD, HA_136-156_ 146N, 10.77% ± 0.72%; HA_143-155_ 146N, 43.78% ± 1.34%) (Fig 2, S19 Table).

### Anti-HCRTR2 autoantibodies were rarely found in narcolepsy patients and controls

**Fig 3:**
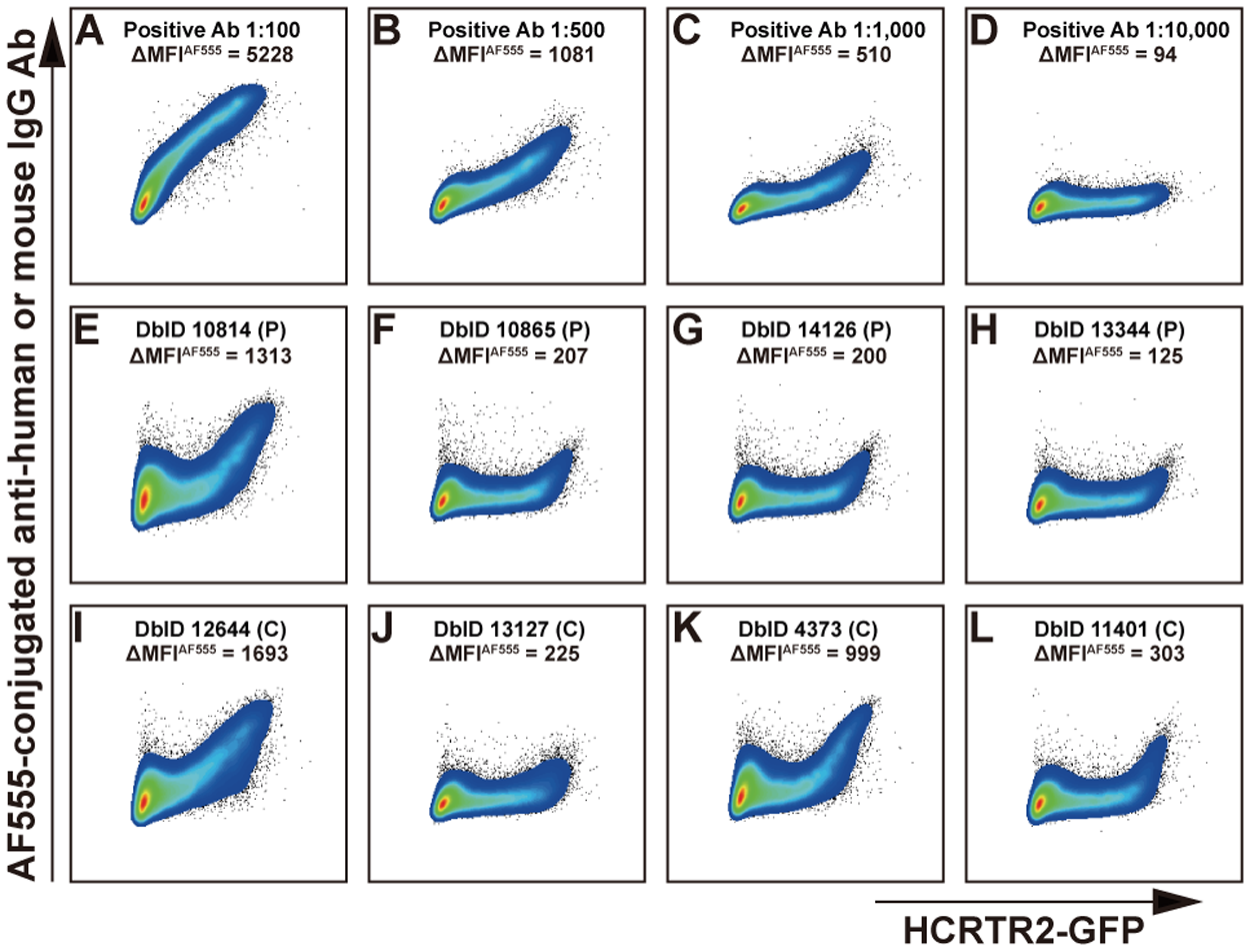
Anti-HCRTR2 autoantibody detection in human sera using flow cytometry. HEK293T cells with transient expression of HCRTR2-GFP were stained with positive anti-HCRTR2 antibodies (Ab) at different dilution ratios (A-D) or human sera (1:20) (E-L), followed by staining with Alexa Fluor^®^ 555 (AF555)-conjugated anti-mouse IgG or anti-human IgG (1:100), respectively. 50,000 events were recorded and dot plots of live single cells were shown with GFP channel (X axis) and AF555 channel (Y axis) for each sample. ΔMFI^AF555^ (mean fluorescence intensity (MFI) of AF555 channel) was determined by subtracting MFI^AF555^ of HEK293T^GFP-^ from MFI^AF555^ of HEK293T^GFP+^. Database identity (DbID) of each subject was also shown. P, patient; C, control.

To detect anti-HCRTR2 autoantibodies in narcolepsy patients and controls, live HEK293T cells transiently transfected with HCRTR2-GFP were stained with each serum from 88 narcolepsy patients, including 40 post Pandemrix^®^ narcolepsy (PP-N) patients and 48 early onset (EO-N) patients, and 88 healthy controls, including 18 matched post Pandemrix^®^ controls (PP-C) and 70 other controls (O-C), and analyzed with flow cytometry. As shown in Fig 3A, the positive anti-HCRTR2 antibody bound to the extracellular domain of HCRTR2 and gave a strong staining signal using Alexa Fluor^®^ 555 (AF555) channel (Fig 3A). For HEK293T cells stained with positive anti-HCRTR2 antibodies, Δ mean fluorescence intensity (MFI) of AF555 channel (ΔMFI^AF555^), which was determined by subtracting MFI^AF555^ of HEK293T^GFP-^ population from MFI^AF555^ of HEK293T^GFP+^ population, was positively correlated with the concentration of positive anti-HCRTR2 antibody (Fig 3A–3D, S1 Fig, S2 Table). Among the 176 subjects tested (S6 and S7 Figs, S20 Table), three subjects showed high ΔMFI^AF555^ (repeated twice) (S1 and S8 Figs, S2 and S21 Tables), which were higher than or comparable to that of positive anti-HCRTR2 antibody at 1:500 dilution (ΔMFI^AF555^, 1081), including one patient (database identity (DbID), 10814, ΔMFI^AF555^, 1313) (Fig 3E, S20 Table) and two controls (DbID, 12644, ΔMFI^AF555^, 1693; DbID, 4373, ΔMFI^AF555^, 999) (Fig 3I and 3K, S20 Table), suggesting presence of anti-HCRTR2 autoantibodies in these three sera. Low ΔMFI^AF555^ was also observed in five other subjects (Fig 3F–3H, 3J and 3L, S20 Table), with lower ΔMFI^AF555^ than that of positive anti-HCRTR2 antibody at 1:1,000 dilution (ΔMFI^AF555^, 510), suggesting lower concentrations of anti-HCRTR2 autoantibodies in these five sera. No positive AF555 signals of HEK293T^GFP+^ population were observed when HEK293T cells were transfected with GFP alone and stained with positive anti-HCRTR2 antibodies or these eight sera (S9 Fig).

### Differential analysis of anti-HCRTR2 autoantibody between narcolepsy patients and controls

**Fig 4:**
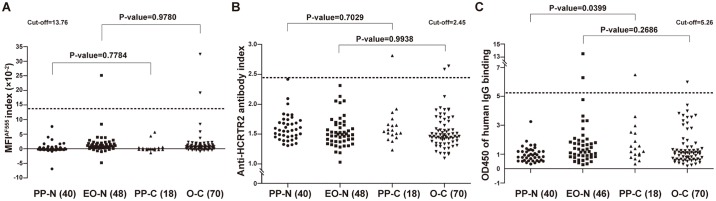
Differential analysis of anti-HCRTR2 autoantibodies using three distinct strategies. Two groups of narcolepsy patients and control subjects were tested using flow cytometry (A), [^35^S]-radiolabelled HCRTR2 binding assay (B), and in-cell ELISA using CHO-HCRTR2 (C). Each dot corresponded to one patient or control subject. The dotted line denoted cut-off value, the mean ± 3× SD of all healthy control subjects for each method. Values above this threshold were considered positive for anti-HCRTR2 autoantibody reaction. PP-N, post Pandemrix^®^ narcolepsy patients; EO-N, early onset narcolepsy patients; PP-C, post Pandemrix^®^ control subjects; O-C, other controls, matched to early onset subjects. The numbers of each group was given. P-values were shown between PP-N and PP-C, and between EO-N and O-C.

To further analyze potential differences in anti-HCRTR2 autoantibody concentrations between narcolepsy patients and controls, the MFI^AF555^ index used to quantify anti-HCRTR2 autoantibodies for each subject was calculated as follows: MFI^AF555^ index = 100× (ΔMFI^AF555^ of subject)/ (ΔMFI^AF555^ of positive anti-HCRTR2 antibody at 1:100 dilution) (S2, S20 and S21 Tables). There was no significant difference regarding the average MFI^AF555^ indexes between narcolepsy patients (n = 88, mean ± SD, 1.01 ± 3.20) and healthy controls (n = 88, mean ± SD; 1.36 ± 4.13) (P-value = 0.5339) (Panel A in S10 Fig, S20 Table). Above the arbitrary cut-off value (13.76), which was determined as Tanaka *et al*. publication [56], positive staining was found in one EO-N patient (MFI^AF555^ index, 25.11; Figs 3E and 4A, S20 Table) and two O-C controls (MFI^AF555^ indexes, 32.38, 19.11; Figs 3I, 3K and 4A, S20 Table), none in post Pandemrix^®^ narcolepsy patients or controls(Fig 4A, S20 Table).

### Anti-HCRTR2 autoantibody detection using [^35^S]-radiolabelled HCRTR2 and in-cell ELISA

We further tested anti-HCRTR2 autoantibodies with the same samples using both [^35^S] radiolabelled HCRTR2 binding assay and in-cell ELISA with CHO-HCRTR2 cell line. No significant difference was found between narcolepsy patients and controls either in the mean of anti-HCRTR2 autoantibody index (p-value = 0.8149; mean ± SD: patients, 1.57 ± 0.24; controls, 1.56 ± 0.30) (Panel B in S10 Fig, S3 Table) with repeat (88 subjects) (S22 Table) or in the corrected OD450 of human IgG binding (p-value = 0.7335; mean ± SD: patients, 1.44 ± 1.63; controls, 1.51 ± 1.25) (Panel C in S10 Fig, S7 Table) with repeat (S8 Table). Three controls showed positive reactions with higher anti-HCRTR2 autoantibody index above cut-off value (2.45), including one PP-C control (autoantibody index, 2.81) and two O-C controls (autoantibody indexes. 2.59, 2.64), and all narcolepsy patients showed negative reactions (Fig 4B, Panel B in S10 Fig, Table 3, S3 Table). Also, positive reactions of human IgG binding to HCRTR2 were detected within two EO-N patients (OD450, 6.29, 13.51), one PP-C control (OD450, 6.49), and one O-C control (OD450, 5.98), accorded to the cut-off value (5.26) (Fig 4C, Panel C in S10 Fig, Table 3, S7 Table).

**Table 3:** Information of positive subjects with at least one test.

DbID	Dx	Gender	Age	DQ0602	HCRT	Interval	Px	MFI^AF555^ index	[^35^S] index	OD450
12644	C	F	16.8	1	NA	NA	N	32.38	1.78	1.01
10814	P	M	12.9	1	NA	0.9	N	25.11	1.50	0.77
4373	C	M	17.7	1	NA	NA	N	19.11	1.16	0.67
11245	C	F	18.6	1	NA	NA	N	-0.49	2.64	1.09
12591	C	F	18.6	1	NA	NA	N	-0.59	2.59	3.16
15053	C	F	10.5	1	NA	NA	Y	0.20	2.81	6.49
12488	P	M	15.1	1	60.63	0.4	N	0.36	1.32	13.51
12264	P	F	5.0	1	0	0.8	N	1.07	2.05	6.29
10940	C	F	20.7	1	NA	NA	N	1.18	1.49	5.98
Cut-off								13.76	2.45	5.26

DbID, database identity; Dx, diagnosis; DQ0602, DQ0602 positive or not; HCRT, hypocretin (HCRT) levels in cerebrospinal fluid (CSF); Interval, month(s) from onset to blood drawn; Post/Px, Pandemrix^®^ vaccined or not; MFI^AF555^ index, index of mean fluorescence intensity of Alexa Fluor^®^ 555 channel; [^35^S] index, index of immune complex of [^35^S]-radiolabelled-HCRTR2 and autoantibody; OD450, optical density at 450 nm of human IgG binding to HCRTR2 in in-cell ELISA. C, control; P, patient; F, female; M, male; Yes, positive; No, negative; NA, not applicable. The cut-off value for a positive sample is determined as the mean + 3× SD of control samples [56].

No positive reaction was found in PP-N patients with each of these three different methods (Fig 4, Table 3, S3, S7 and S20 Tables). No significant difference was found either between PP-N patients and matched PP-C controls or between EO-N patients and matched O-C controls with each of the three tests, except for the former pairs in in-cell ELISA (p-value = 0.0399) (Fig 4, S3, S7 and S20 Tables). Although no a single subject showed positive results with all the three different tests, one PP-C control (DbID 15053) was found positive in both [^35^S]-radiolabelled HCRTR2 binding assay and In-Cell ELISA, but not in flow cytometry analysis (Fig 4, Table 3, S3, S7 and S20 Tables). Any other subject which showed positive reaction with one test was found negative with other two methods (Fig 4, Table 3, S3, S7 and S20 Tables). No known factor was positively correlated to these positive subjects (Table 3).

## Discussion

This work shows that anti-HCRTR2 autoantibodies were rarely detected (3 or 4 positive reactions of 176 sera), with no positive subject found in post Pandemrix^®^ narcolepsy patients (PP-N). We were unable to confirm that anti-HCRTR2 autoantibodies were present in a large portion of the population, extending on the work in 191 samples recently published by Giannoccaro *et al*. [61], who used HEK293 cells transiently transfected with human HCRTR2 and scored each serum stained with HEK293 cells, and found 3 of 61 patients positive: two type 1 narcolepsy and one type 2 narcolepsy, but none of the control subjects. In our study, to maximize the possibility of finding positives, we tested 40 post Pandemrix^®^ narcolepsy cases and 18 matched controls (similar to those used in the Ahmed *et al*. [43]), plus 48 sera of patients who had a very recent onset (≤6 months) of narcolepsy. The last group was selected because of the highest likelihood the autoimmune process would still have been active in these patients, and thus detection of autoantibody would be the easiest. With CBA detection described by Giannoccaro *et al*. [61], positive staining was not observed with positive anti-HCRTR2 antibody (S11 Fig). Therefore, detection of anti-HCRTR2 autoantibodies was performed using three other independent techniques, one of which was similar to that used by Ahmed *et al*. [43] (in-cell ELISA) except for the use of a different cell line, CHO-K1 instead of Chem-1. The use of the CHO-K1 cell line was due to the fact that the stably transfected Chem-1-HCRTR2 cell line could not be made available to us. The second technique, [^35^S]-radiolabelled HCRTR2 binding assay, had been previously reported by Tanaka *et al*. [56], who could not find evidence for anti-HCRTR2 autoantibodies. Using this second technique, we confirmed this observation (Fig 4, Table 3, S10 Fig, S3 and S22 Tables). The disadvantage of this method was that *in vitro* translated polypeptide chain was unlikely to be conformationally similar to HCRTR2 that was naturally embedded in cytoplasmic membranes. Because of the limitations of these two techniques (different cell line and conformational difference), we also used a third, more novel technique, which involved HEK293T cells transiently transfected with HCRTR2-GFP. Cells that were double labeled with GFP and anti-HCRTR2 antibody could be easily visualized in the upper right quadrant and distinguished from background or cells that have not successfully expressed the transgene. This last method, which has to our knowledge only been used by a few investigators, such as for the detection of MUC1-Tn antibodies [55], or N-methyl-D-aspartate receptor (NMDAR) or dopamine-2 receptor (D2P) autoantibodies [54], was found to be more sensitive (see positive controls in Fig 3 and S1 Fig) and yielded a fewer positives in both narcolepsy patient and control subjects (Fig 3, Table 3, S1 Fig, S2 Table). Like the in-cell ELISA, this last technique should be targeting a normally conformed HCRTR2. As noted in Table 3, a few positives were found with one or another technique, but none of the sera reacted positively using all the three techniques.

Although these results were disappointing, it was also notable that the presence of anti-HCRTR2 autoantibodies in some narcolepsy patients and controls reported by Ahmed *et al*. [43] was unlikely to be causative to the condition. Indeed, in narcolepsy, the cells containing the hypocretin ligand, not those carrying hypocretin receptors, were the targets of the autoimmune process. Hypocretin receptors were expressed on a large number of target neurons in the brain [62], and one would have had to hypothesize some special vulnerability for hypocretin cells to be destroyed by an anti-HCRTR2 process. Further, as discussed by Vassali and Tafti [63], hypocretin receptors are likely not present on hypocretin cells.

This report also allowed for the clarification of the HA sequence at position 146 and NP sequence at position 116 in these vaccines, and the ability of these sequences to bind to DQ0602. Regarding HA 146N, the fact that the pH1N1 wild-type sequence HA_143-155_ (HDSNKGVTAACPH) (146N was underlined) was present in Pandemrix^®^, Arepanrix^®^ and Focetria^®^ was difficult to interpret with respect to narcolepsy risk. Our peptide binding studies confirmed that HA_136-156_ (KTSSWPNHDSNKGVTAACPHA) containing 146N (underlined) bound to DQ0602, and that the introduction of the D mutation at 146 position reduced binding affinity (Fig 2 and S19 Table), a result that was predicted by bioinformatics in our prior publication [33]. Surprisingly, however, whereas information provided by Novartis has indicated that Focetria^®^ used in the vaccination campaign was primarily derived from X-181, in this study we found that the predominant residue at 146 position was N (Table 2), like Pandemrix^®^, suggesting the strain used to produce this particular batch was X-179A, not X-181. Further examination of EMA records indicated that a switch to X-181 likely operated in the midseason of 2009 as use was originally granted for A/California/07/2009 (H1N1)v-like strain NYMC X-179A, while X-181 use was approved on 11/11/2009 (see EMA information at http://www.ema.europa.eu/ema/index). It was thus likely that the batch in this study provided by Novartis was an earlier batch, and that most of the pH1N1 vaccination campaign used X-181 and the HA_143-155_ (HDSDKGVTAACPH) sequence containing 146D (underlined), differently from the batch examined here. The hypothesis that HA_136-156_ (KTSSWPNHDSNKGVTAACPHA) containing 146N (underlined), a DQ0602 binding epitope predominantly present in Pandemrix^®^ and the pH1N1-2009 virus may be related to narcolepsy risk remained a possibility. In spite of multiple efforts, however, we have been unable to find homology for this sequence with known hypocretin neuron proteins [64–66].

Regarding the NP 116I, unlike in the wild-type PR8 NP (H1N1-1918-like) sequence, it appeared that this residue has been mutated from I to M in the X-179A strain that has been used for production of not only Pandemrix^®^, but also Arepanrix^®^ and Focetria^®^. The fact that NP_111-121_ (YDKEEIRRIWR) (116I was underlined) and HCRTR2_34-45_ (YDDEEFLRYLWR) did not appear to bind to DQ0602 (unlike what was reported with the Proimmune^®^ array by Ahmed *et al*. [43]) (Fig 2, S19 Table) suggested this epitope was likely irrelevant to DQ0602-associated narcolepsy or differential vaccine risk. Regarding the antibody cross-reactivity previously reported with this epitope by Ahmed *et al*. [43], and the fact that could not be reproduced here, explanations could be advanced. Weak antibody cross-reactivity between infectious targets and self-proteins may not be as rare as anticipated. For example, a few years ago, in a study of anti-streptococcal antibodies in narcolepsy, we found that anti-streptolysin (ASO) antibodies, observed more frequently in recent onset narcolepsy sera, cross-reacted weakly with the human phosphodiisomerase (PDI) protein [14, 67]. This result was initially exciting to us, however, we rapidly found these autoantibodies to be present in both ASO-positive narcolepsy patients and controls tempering our enthusiasm. Similarly, Deloumeau *et al*. [68] reported autoantibodies against hypocretin instead of the HCRTR2 as immune complexes, observations that were only seen in post chemical treatment sera, a finding that has not been reproduced to date.

In conclusion, in this comprehensive study we confirmed that mutations could accumulate in specific flu vaccines as they were propagated in eggs, deviating from the original strain sequence. The presence of one specific mutation in Arepanrix^®^, HA 146D, was confirmed in multiple batches used in Canada. Unfortunately, we were not able to use those batches that were used in Scandinavia in 2009–2010. Theoretically there was a possibility that they may have differed from the batches we studied. Skowronski *et al*. [69] similarly found that key antigenic residues were mutated in H3N2 reassortant vaccine strains and that this likely contributed to reduced efficacy in 2012–2013. These results suggest that regular DNA sequencing or MS characterization of viral isolates may be useful as quality control measures across long periods of production. We also found that previously reported results suggesting molecular mimicry between NP and HCRTR2 could not be reproduced and that autoreactivity by autoantibodies to HCRTR2 was unlikely to play a role in the pathophysiology of narcolepsy.

## Supporting information

S1 Raw Data MS raw data of Pandemrix^®^ and Arepanrix^®^. 5 “.byrslt” format files are included and can be opened with Byonic^®^. Representative spectrum of HA 146N, HA 146D, or NP 116I was exported from the file with the corresponding specific name.
(RAR)

S2 Raw Data MS raw data of Pandemrix^®^, Arepanrix^®^ and Focetria^®^. 5 “.byrslt” format files are included and can be opened with Byonic^®^. Representative spectrum of HA 146N, HA 146D, NP 116I, or NP 116M was exported from the file with the corresponding specific name.
(RAR)

S1 Fig Staining sensitivity of positive anti-HCRTR2 antibody and staining of sera (first repeat) with flow cytometry. HEK293T cells with transient expression of HCRTR2-GFP were stained with positive anti-HCRTR2 antibodies (Ab) at different dilution ratios (1:100,000 to 1:20) or human sera (1:20), followed by staining with Alexa Fluor^®^ 555 (AF555)-conjugated anti-mouse IgG or anti-human IgG (1:100), respectively. Dot plots of live single cells are shown with GFP channel (X axis) and AF555 channel (Y axis) for each sample with database identity (DbID). HEK293T cells stained with only AF555-conjugated anti-mouse IgG (Anti-mouse IgG) or anti-human IgG (Anti-human IgG) (1:100), or without any antibody staining (HCRTR2-GFP) are shown as background control.
(TIF)

S2 Fig Detection of [^35^S]-radiolabelled HCRTR2 in elution fractions. [^35^S]-radiolabelled HCRTR2 was synthesized *in vitro* using TNT^®^ quick coupled transcription/translation system according to the manufacturer’s instructions. The reaction mixture was loaded into an illustra^®^ NICK^®^ column. Equal volume of eight elution fractions was loaded into a precast protein gel. Radioactive exposure of [^35^S]-radiolabelled HCRTR2 (arrow) and protein marker ladder are shown.
(TIF)

S3 Fig Higher expression of transgene HCRTR2 in CHO-HCRTR2 cell line with flow cytometry. Host cells CHO-K1 (black line) and transgenic cells CHO-HCRTR2 (red line) were stained with positive primary polyclonal goat anti-HCRTR2 antibody (A) or monoclonal mouse anti-HCRTR2 antibody (B) (1:100), followed by secondary Alexa Fluor^®^ 555 (AF555)-conjugated anti-goat IgG or anti-mouse IgG (1:100), respectively. CHO-K1 (grey line) and CHO-HCRTR2 (blue line) staining with only secondary AF555-conjugated anti-goat IgG or anti-mouse IgG (1:100) are shown as controls.
(TIF)

S4 Fig Detection of HCRTR2 expression using western blotting. 8.163 millions of live CHO-K1 or CHO-HCRTR2 cells were lysed in RIPA buffer. Equal volume of whole cell lysates were loaded into precast protein gel. Blot was incubated with mouse monoclonal anti-HCRTR2 antibody (1:500), followed by incubation secondary peroxidase-conjugated anti-mouse IgG (H+L) antibody (1:5000 dilution). HCRTR2 is detected as indicated (arrow).
(TIF)

S5 Fig Representative MS spectra of HA peptides around HA 146N or 146D as observed in Pandemrix^®^ (A and B), Arepanrix^®^ (C and D), and Focetria^®^ (E and F). Series a/b and y of peptides are numbered from N-terminal and C-terminal ends, respectively. Associated series number denoted size of fragments in amino acid residue number, from 1 to 10. HA 146N to 146D mutation corresponded to a one Da shift.
(TIF)

S6 Fig Dot plots of serum (location L-A01 to L-H12) staining with flow cytometry. HEK293T cells with transient expression of HCRTR2-GFP were stained with positive anti-HCRTR2 antibodies (Ab) (1:100) or human sera (1:20), followed by staining with Alexa Fluor^®^ 555 (AF555)-conjugated anti-mouse IgG or anti-human IgG (1:100), respectively. Dot plots of live single cells are shown with GFP channel (X axis) and AF555 channel (Y axis) for each sample with database identity (DbID). HEK293T cells stained with only AF555-conjugated anti-mouse IgG (Anti-mouse IgG) or anti-human IgG (Anti-human IgG) (1:100), or without any antibody staining (HCRTR2-GFP) are shown as background control.
(TIF)

S7 Fig Dot plots of serum ((location G-A05 to G-H12) staining with flow cytometry. HEK293T cells with transient expression of HCRTR2-GFP were stained with positive anti-HCRTR2 antibodies (Ab) (1:100) or human sera (1:20), followed by staining with Alexa Fluor^®^ 555 (AF555)-conjugated anti-mouse IgG or anti-human IgG (1:100), respectively. Dot plots of live single cells are shown with GFP channel (X axis) and AF555 channel (Y axis) for each sample with database identity (DbID). HEK293T cells stained with only AF555-conjugated anti-mouse IgG (Anti-mouse IgG) or anti-human IgG (Anti-human IgG) (1:100), or without any antibody staining (HCRTR2-GFP) are shown as background control.
(TIF)

S8 Fig Sera staining of replicates with flow cytometry. HEK293T cells with transient expression of HCRTR2-GFP were stained with positive anti-HCRTR2 antibodies (Ab) (1:100) or human sera (1:20), including potential positive sera according to results of the first screening, followed by staining with Alexa Fluor^®^ 555 (AF555)-conjugated anti-mouse IgG or anti-human IgG (1:100), respectively. Dot plots of live single cells are shown with GFP channel (X axis) and AF555 channel (Y axis) for each sample with database identity (DbID). HEK293T cells stained with only AF555-conjugated anti-mouse IgG (Anti-mouse IgG) or anti-human IgG (Anti-human IgG) (1:100), or without any antibody staining (HCRTR2-GFP) are shown as background control.
(TIF)

S9 Fig Staining controls of HEK293T cells expressing GFP alone using flow cytometry. HEK293T cells with transient expression of GFP alone were stained with positive anti-HCRTR2 antibody at different dilution ratios (A and B) or human serum (1:20) (E-L), followed by staining with Alexa Fluor^®^ 555 (AF555)-conjugated anti-mouse IgG or anti-human IgG (1:100), respectively. Dot plots of live single cells are shown with GFP channel (X axis) and AF555 channel (Y axis) for each sample with database identity (DbID). HEK293T cells stained with only AF555-conjugated anti-mouse IgG (C) or anti-human IgG (D) (1:100) are shown as background control.
(TIF)

S10 Fig Differential analysis of anti-HCRTR2 autoantibodies between narcolepsy patients and controls. Narcolepsy patients and controls were tested using flow cytometry (A), [^35^S]-radiolabelled HCRTR2 binding assay (B), and in-cell ELISA using CHO-HCRTR2 (C). Each dot corresponds to one patient or a control subject. The dotted line denotes the cut-off value, the mean ± 3× SD of all healthy control subjects for each method. Values above this threshold were considered positive for anti-HCRTR2 autoantibody reaction. The numbers of each group is given. P-values are shown between patients and controls.
(TIF)

S11 Fig Fluorescence images of positive anti-HCRTR2 antibody staining. HEK293T cells transiently expressing HCRTR2-GFP were stained with positive anti-HCRTR2 antibody (1:100), followed by secondary Alexa Fluor^®^ 555 (AF555)-conjugated antibody (1:1000), GFP channel (A), AF555 channel (B), DAPI channel (C), and overlay (D) are shown. Bar = 200 μm.
(TIF)

S1 Table Participant information, including gender, age, DQ0602, Pandemrix^®^ vaccination and diagnosis. (XLSX)

S2 Table Raw data of positive anti-HCRTR2 antibody staining sensitivity and serum staining with flow cytometry for first repeat, corresponding to S1 Fig. (XLS)

S3 Table Raw data of anti-HCRTR2 autoantibody analysis using [^35^S]-radiolabelled HCRTR2 binding assay, corresponding to Fig 4B and Panel B in S10 Fig. (XLSX)

S4 Table Raw data of cell number per well (625–10,000) analysis for in-cell ELISA. (XLSX)

S5 Table Raw data of cell number per well (10,000–100,000) analysis for in-cell ELISA. (XLSX)

S6 Table Raw data of two positive anti-HCRTR2 antibodies in rabbit sera test using in-cell ELISA. (XLSX)

S7 Table Raw data of anti-HCRTR2 autoantibody analysis using in-cell ELISA, corresponding to Fig 4C and Panel C in S10 Fig. (XLSX)

S8 Table Raw data of anti-HCRTR2 autoantibody analysis using in-cell ELISA for repeat. (XLSX)

S9 Table MS details of peptide fragment around NP 116M from Pandemrix^®^ corresponding to S2 Raw Data. (XLSX)

S10 Table MS details of peptide fragment around NP 116M from Arepanrix^®^ corresponding to S2 Raw Data. (XLSX)

S11 Table MS details of peptide fragments around NP 116M, HA 146N, and HA 146D from Focetria^®^ corresponding to S2 Raw Data. (XLSX)

S12 Table MS details of peptide fragment around NP 116I from Pandemrix^®^ corresponding to S2 Raw Data. (XLSX)

S13 Table MS details of peptide fragment around NP 116I from Arepanrix^®^ corresponding to S1 Raw Data. (XLSX)

S14 Table MS details of peptide fragments around NP 116I and HA 146N from Pandemrix^®^ corresponding to S2 Raw Data. (XLSX)

S15 Table MS details of peptide fragment around HA 146N from Arepanrix^®^ corresponding to S1 Raw Data. (XLSX)

S16 Table MS details of peptide fragment around HA 146N from Arepanrix^®^ corresponding to S1 Raw Data. (XLSX)

S17 Table MS details of peptide fragment around HA 146D from Pandemrix^®^ corresponding to S1 Raw Data. (XLSX)

S18 Table MS details of peptide fragment around HA 146D from Arepanrix^®^ corresponding to S1 Raw Data. (XLSX)

S19 Table Raw data of peptide binding to DQ0602, corresponding to Fig 2. (XLSX)

S20 Table Raw data of anti-HCRTR2 autoantibody analysis using flow cytometry, corresponding to Fig 4A and Panel A in S10 Fig. (XLSX)

S21 Table Raw data of serum staining with flow cytometry for second repeat, corresponding to S8 Fig. (XLS)

S22 Table Raw data of anti-HCRTR2 autoantibody analysis using [^35^S]-radiolabelled HCRTR2 binding assay for repeat. (XLSX)
