# Correction: Absence of anti-hypocretin receptor 2 autoantibodies in post pandemrix narcolepsy cases

**DOI:** 10.1371/journal.pone.0214340

**Published:** 2019-03-28

**Authors:** Guo Luo, Ling Lin, Louis Jacob, Mélodie Bonvalet, Aditya Ambati, Giuseppe Plazzi, Fabio Pizza, Ryan Leib, Christopher M. Adams, Markku Partinen, Emmanuel Jean-Marie Mignot, and the PLOS ONE Editors

Following publication of this [1] article, questions were raised about some of the reported methods and results, and about differences between the findings reported in this article and in a previous article published by another group [2]. The *PLOS ONE* Editors reviewed this matter, and consider that the research reported in the *PLOS ONE* article is scientifically valid and meets the journal’s publication criteria, but that some items require clarification and additional controls. The authors address these issues below:

“As noted in [1], we were unable to replicate some findings reported by Ahmed et al. in [2]. We would like to provide some clarifications regarding some methodological differences between the two studies.

First, a statement in the Discussion was inaccurate in relaying the differences between the results of peptide binding affinity to DQ0602. The statement: ‘The fact that NP_111-121_ (YDKEEIRRIWR) (116I was underlined) and HCRTR2_34-45_ (YDDEEFLRYLWR) did not appear to bind to DQ0602 (unlike what was reported with the Proimmune^®^ array by Ahmed et al. [43]) (Fig 2, S19 Table) suggested this epitope was likely irrelevant to DQ0602-associated narcolepsy or differential vaccine risk’ should instead read: The fact that NP_111-121_ (YDKEEIRRIWR) (116I was underlined) and HCRTR2_34-45_ (YDDEEFLRYLWR) did not appear to bind to DQ0602 (Competition binding results S23 Table this Correction) (Of note, ‘NP109-113 116I’ of Fig 2 in [1] should be corrected to ‘NP109-123 116I’) suggested this HCRTR2 epitope was likely irrelevant to DQ0602-associated narcolepsy or differential vaccine risk. Indeed, S3 Table of Ahmed and Steinman’s Proimmune REVEAL^®^ data [2] showed weak binding for RELILYDKEEIRRIWRQANNG (24.4 and 16.9 of REVEAL^®^ score at first measure and post 24h, respectively), little binding for RELILYDKEEMRRIWRQANNG (1.5 and 0.5 of REVEAL^®^ score at first measure and post 24h, respectively) and no binding for the HCRTR2 peptide (LNPTDYDDEEFLRYLWREYLH) (0.0 of REVEAL^®^ score at both first measure and post 24h). We overlooked these small differences. Our results show little binding for RELILYDKEEIRRIWRQANNG (92.40±2.33% of Bio-EBV binding), very weak binding for RELILYDKEEMRRIWRQANNG (77.84±1.10% of Bio-EBV binding) and no binding for the HCRTR2_34-45_ peptide (98.12±1.38% or 99.85±2.10% of Bio-EBV binding) (competition binding results S23 Table this Correction). This does not change our interpretation.

Second, intrigued by the results reported by Ahmed et al. [2], we also conducted our own peptide binding studies to DQ0602 using the Proimmune REVEAL^®^ binding assay (See S1 File of this Correction) and compared these results for 144 peptides with our own competition assay that uses DQ0602 monomers bound to Bio-EBV (more than 1,446 peptides have been tested using this platform). Results are shown in S12 Fig this Correction S23 Table this Correction; underlying data and analyses are provided in S2 File of this Correction. Overall, no significant correlation was found between our competition binding assay using Bio-EBV and Proimmune results (r^2^ = 0.00795, p = 0.290 at 0h; r^2^ = 0.00026, p = 0.848 at S12 Fig this Correction). Classifying our binders into strong (high displacement, ≤25% of Bio-EBV binding) or weak binders (partial displacement, 25–50% of Bio-EBV binding) in our competition assay and positive versus negative in the Proimmune assay also did not reveal any correlation using χ^2^ (all p values>0.56, S2 File of this Correction). Further illustrating this, the known EBV binding epitope of DQ0602 (EBV_486-500_) [6–8], which was used in our competition binding assay, and HA_273-287_, a strong binder in our assay (8.10±1.53% of Bio-EBV S23 Table this Correction), were found not to bind to DQ0602 using the Proimmune^®^ assay (0.0 of REVEAL^®^ score at both 0h and post 24h, S1 File of this Correction). This led us to conclude that the Proimmune DQ0602 binding assay was unreliable. However, as these results were peripheral to the message of our manuscript and lack of replication of the presence of anti-HCRTR2 antibodies, these were not included in our original publication.

Third, in anti-HCRTR2 antibody testing experiments using serum samples presented in Luo et al. [1], when we wrote we tested ‘similar’ samples to Ahmed et al. [2], we did not mean to indicate we tested the exact same sera as Ahmed et al. [2]. We meant to write that we tested ‘similar’ post-Pandemrix^®^ narcolepsy and control samples but collected in other countries (Ireland, primarily) (instead of Finland for Ahmed et al. [2]). Our results with these samples (Fig 4 in [1]) differ from what is reported in Ahmed et al. (Fig 2 in [2]). Unfortunately, we could not repeat testing of post-Focetria^®^ control (non-narcolepsy) samples, as these come from a clinical trial and are proprietary to Novartis^®^. In Ahmed et al. [2], none of the post-Focetria^®^ samples were reported to have anti-HCRTR2 antibodies, unlike narcolepsy and control and samples from Finland which are positive. We found that Focetria^®^ and Pandemrix^®^ do not differ significantly in their NP sequence [1] suggested to be homologous to HCRTR2.

To further clarify the similarities in samples used in the two studies and enable future efforts toward replicating these results, we provide the following details regarding participant selection for the study reported in [1]. Inclusion criteria for type 1 narcolepsy were narcolepsy with cataplexy and HLA-DQ0602 positivity, and/or documented low CSF hypocretin-1. These criteria are based on the ICSD-3 and DSM-5 guidelines for diagnosing narcolepsy [4]. Controls were either healthy spouse or friends of patients with narcolepsy or with other sleep disorders. These were screened for the absence of narcolepsy through interviews and using a questionnaire [5] surveying symptoms for all sleep disorders that contains questions about narcolepsy symptoms. The patients selected for the Ahmed et al. [2] study were selected on the basis of similar clinical criteria.

Fourth, regarding S4 Fig in [1], it was raised that the two lanes had substantial differences in the number and weight of background bands, and that the presented western blot did not clearly show that the transfected cell line had a strong specific band at the molecular weight expected for HCRTR2. S13 Fig this Correction, we now present a repeat of this experiment that also includes a gel image showing the amount of protein extract used in this western blot experiment, plus a higher magnification view of the region involved.

The experiment aimed to provide evidence that the commercial antibody recognized HCRTR2, validating this antibody as positive controls for other assays. Of note, non-specific bands are still observed outside of the focus area. In our opinion, these non-specific bands we have observed are likely due to cross reactivity, post-translation modification of HCRTR2, or fragment, although a monoclonal antibody was used. It is notably difficult to obtain specific anti-G-protein coupled receptor (GPCR) antibodies and the western blot was meant to show that the antibody may have off-target effects but was still targeting HCRTR2, which was sufficient as a positive control for our experiments using FACS [1].

Fifth, due to difficulty obtaining a proprietary cell line, we used alternative cell lines in efforts to replicate the experiments reported in Ahmed et al. [2] and obtained different results as reported in Luo et al. [1]. CHO-K1 cells were purchased in March 2016 from ATCC (https://www.atcc.org/products/all/CCL-61.aspx), and the CHO-HCRTR2 cell line, which was derived from CHO-K1, was purchased prior to 2008 from GenScript (TM0508, GenScript, CHO-K1/OX2). It should be noted that the Chem-1 cell line, which was used in Ahmed et al. [2], is described by the supplier Eurofins Discovery Services as lacking endogenous expression of most GPCRs [3], which may not be the case with CHO-K1. This could explain a very faint band at that location in our control cell line.

Sixth, it was mentioned that in S11 Fig in [1], microscopy revealed HEK293 cells expressing HCRTR2 in green (green fluorescent protein, GFP), mouse monoclonal antibody staining of human HCRTR2 in red (Alexa Fluor 555), and nuclear staining in blue (DAPI) but that the antibody staining against HCRTR2 is not demonstrating the expected “punctate” staining pattern that would be typical for a GPCR such as HCRTR2. This was primarily due to the poor quality of the microscope used. To complement this figure, we have now repeated the experiment using a confocal microscope and show clear punctuate colocalized staining (S14 Fig of this Correction). We are also showing similar punctate staining of HCRTR2 with Alexa Fluor 555 on CHO-HCRTR2 cell line constitutionally expressing HCRTR2, but not in control CHO-K1 (S15 Fig of this Correction).”

The Competing Interests statement for this article is incorrect. The correct statement is: EM currently receives funding from Jazz Pharmaceutical and EM previously received funding from GlaxoSmithKline for the study of the immunological basis of post-Pandemrix^®^-narcolepsy. Conduct for these studies were supervised by and reported to the European Medical Agency. GlaxoSmithKline holds the patent for Pandemrix^®^. Funding from these two sources did not support the research reported in this article [1]. In addition, a provisional patent on a potential DQ0602 hemagglutinin flu epitope sequence cross-reactive with hypocretin was filed by GSK and Stanford with EM as one of the inventors, but the patent was subsequently abandoned when the publication of De la Herran-Arita et al. [9] was retracted [10].

## Supporting information

S12 Fig Lack of correlation of results obtained for DQ0602 binding using our in-house Bio-EBV competition binding assay and the Proimmune REVEAL^®^ assay. 144 peptides were tested using both assays. Note that the Bio-EBV competition results were inverted (100%-displacement) so that higher value indicate higher binding for easier comparison with the Proimmune assay.
(TIF)

S13 Fig Repeat western blot of HCRTR2 over-expressing cell line (CHO-HCRTR2) versus control host cell line (CHO-K1). A. Coomassie blue staining of whole cell lysates used in S4 Fig of Luo et al. [1]. Note equal amount of protein in both lanes. B. Repeat western blot of the same protein lysates stained with monoclonal anti-HCRTR2 antibody (Cat# WH0003062M1-100UG, Sigma), with focus on the area of HCRTR2 molecular weight size.
(TIF)

S14 Fig HEK293T and HCK293T-HCRTR2-GFP cells staining with monoclonal anti-HCRTR2 antibody (Cat# WH0003062M1-100UG, Sigma). Cells were cultured and stained as described in [1] (see “Anti-HCRTR2 autoantibody detection with flow cytometry”). Images were taken using Leica TCS SP8 confocal microscope. Contrast and brightness of the digital image of only DAPI channel from HEK293T-HCRTR2-GFP were slightly adjusted for easier viewing. AF555, Alexa Fluor 555.
(TIF)

S15 Fig CHO-K1 and CHO-HCRTR2 cell lines staining with polyclonal anti-HCRTR2 antibody (Cat# ab65093, Abcam). Cells were cultured and stained as described in [1] (see “Anti-HCRTR2 autoantibody detection using in-cell ELISA”). Images were taken using Leica TCS SP8 confocal microscope. These results complement our in-cell ELISA results obtained with this cell line. AF555, Alexa Fluor 555.
(TIF)

S1 File DQ0602 binding of Proimmune REVEAL^®^ assay. (XLSX)

S2 File Raw data and analyses underlying S12 Fig this Correction. (XLSX)

S3 File Original gel image supporting results in panel A of S13 Fig. (TIF)

S4 File Original blot images supporting results in panel B of S13 Fig. Note that the blot on the left, probed with the monoclonal antibody, was used in preparing S13 Fig.
(TIF)

S23 Table NP and HCRTR2 peptides binding to DQ0602 using Bio-EBV competition assay and Proimmune REVEAL^®^ assay. (XLSX)
