# Supplementary figures and images for: Absence of anti-hypocretin receptor 2 autoantibodies in post pandemrix narcolepsy cases
Source: PLoS One. 2017 Dec 8;12(12):e0187305. doi: 10.1371/journal.pone.0187305 (PMC5722318; doi:10.1371/journal.pone.0187305)

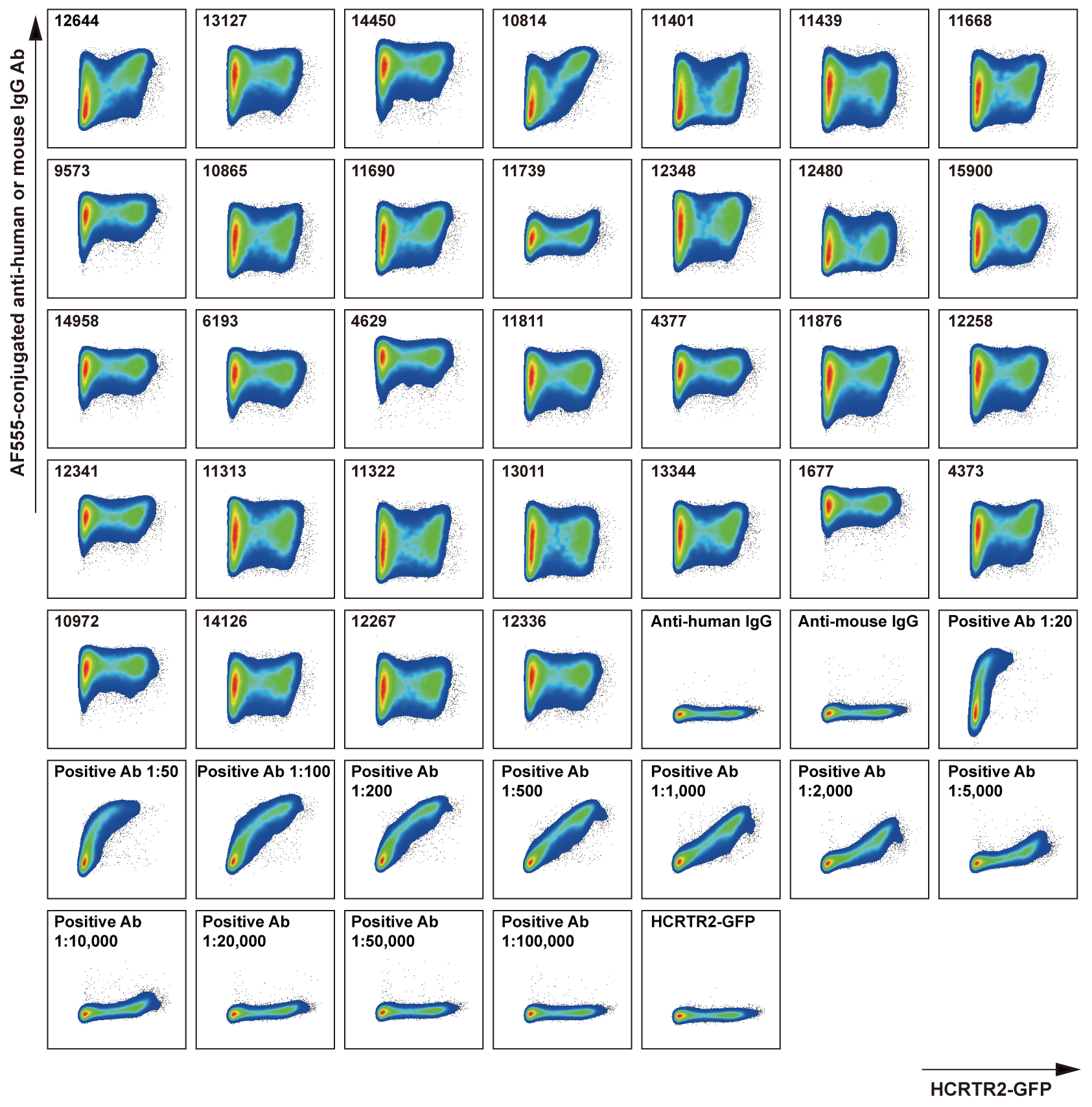

Supplement: S1 Fig — HEK293T cells with transient expression of HCRTR2-GFP were stained with positive anti-HCRTR2 antibodies (Ab) at different dilution ratios (1:100,000 to 1:20) or human sera (1:20), followed by staining with Alexa Fluor® 555 (AF555)-conjugated anti-mouse IgG or anti-human IgG (1:100), respectively. Dot plots of live single cells are shown with GFP channel (X axis) and AF555 channel (Y axis) for each sample with database identity (DbID). HEK293T cells stained with only AF555-conjugated anti-mouse IgG (Anti-mouse IgG) or anti-human IgG (Anti-human IgG) (1:100), or without any antibody staining (HCRTR2-GFP) are shown as background control. (TIF) [file pone.0187305.s003.tif]

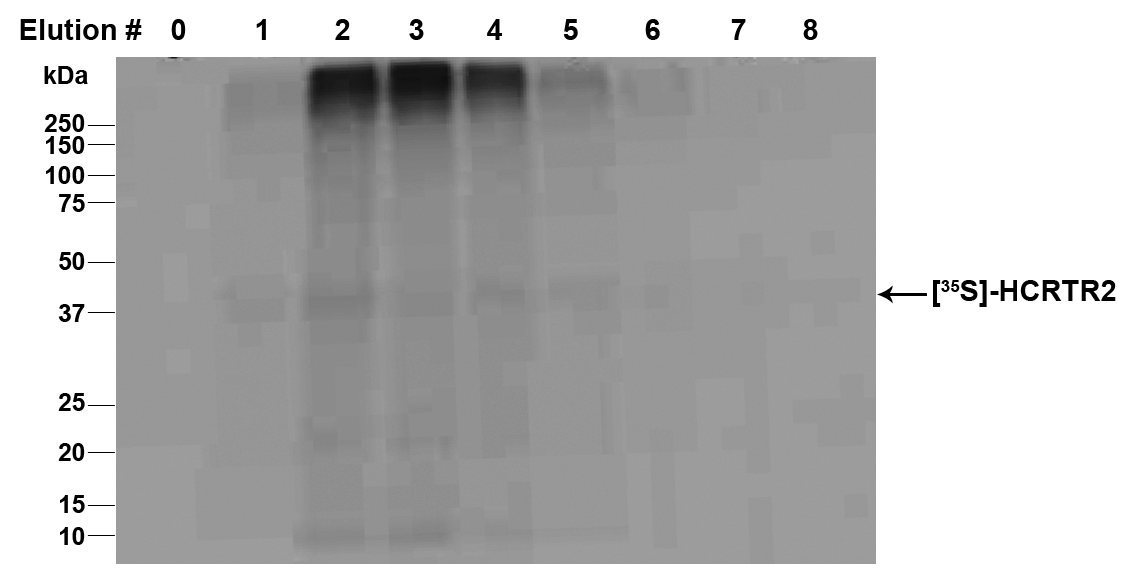

Supplement: S2 Fig — [35S]-radiolabelled HCRTR2 was synthesized in vitro using TNT® quick coupled transcription/translation system according to the manufacturer’s instructions. The reaction mixture was loaded into an illustra® NICK® column. Equal volume of eight elution fractions was loaded into a precast protein gel. Radioactive exposure of [35S]-radiolabelled HCRTR2 (arrow) and protein marker ladder are shown. (TIF) [file pone.0187305.s004.tif]

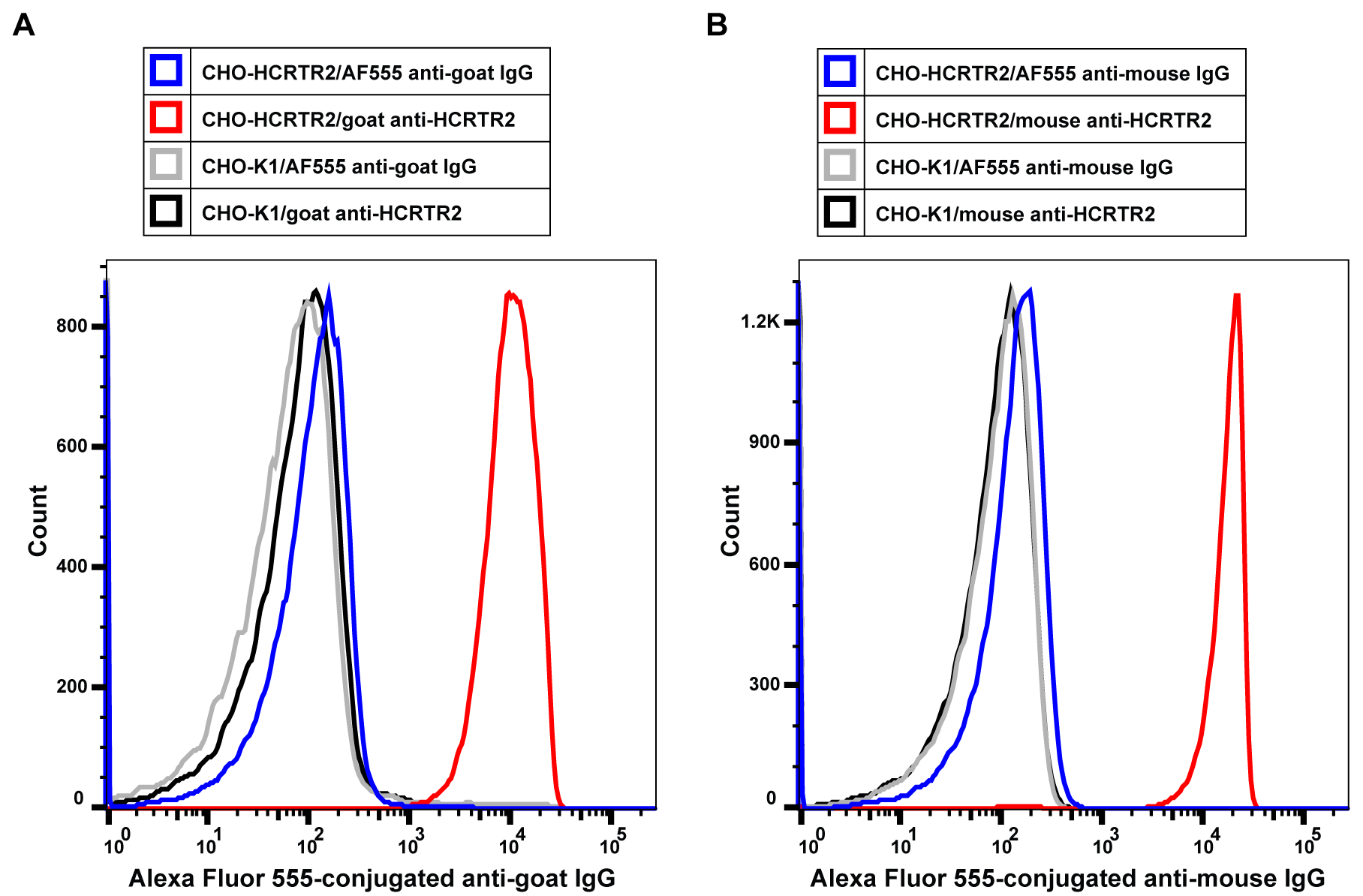

Supplement: S3 Fig — Host cells CHO-K1 (black line) and transgenic cells CHO-HCRTR2 (red line) were stained with positive primary polyclonal goat anti-HCRTR2 antibody (A) or monoclonal mouse anti-HCRTR2 antibody (B) (1:100), followed by secondary Alexa Fluor® 555 (AF555)-conjugated anti-goat IgG or anti-mouse IgG (1:100), respectively. CHO-K1 (grey line) and CHO-HCRTR2 (blue line) staining with only secondary AF555-conjugated anti-goat IgG or anti-mouse IgG (1:100) are shown as controls. (TIF) [file pone.0187305.s005.tif]

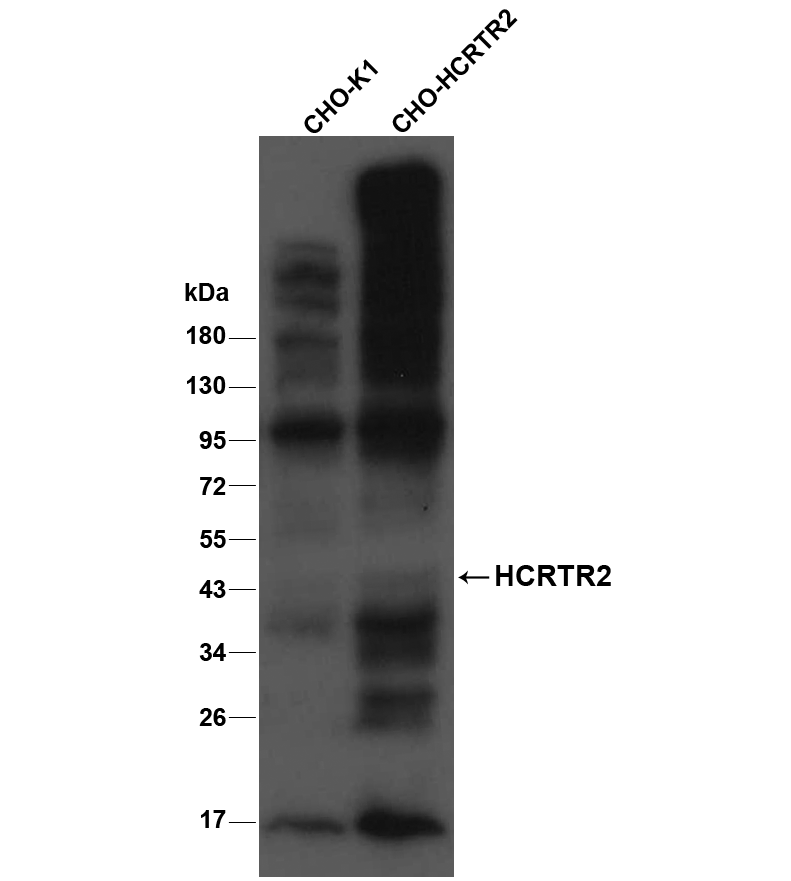

Supplement: S4 Fig — 8.163 millions of live CHO-K1 or CHO-HCRTR2 cells were lysed in RIPA buffer. Equal volume of whole cell lysates were loaded into precast protein gel. Blot was incubated with mouse monoclonal anti-HCRTR2 antibody (1:500), followed by incubation secondary peroxidase-conjugated anti-mouse IgG (H+L) antibody (1:5000 dilution). HCRTR2 is detected as indicated (arrow). (TIF) [file pone.0187305.s006.tif]

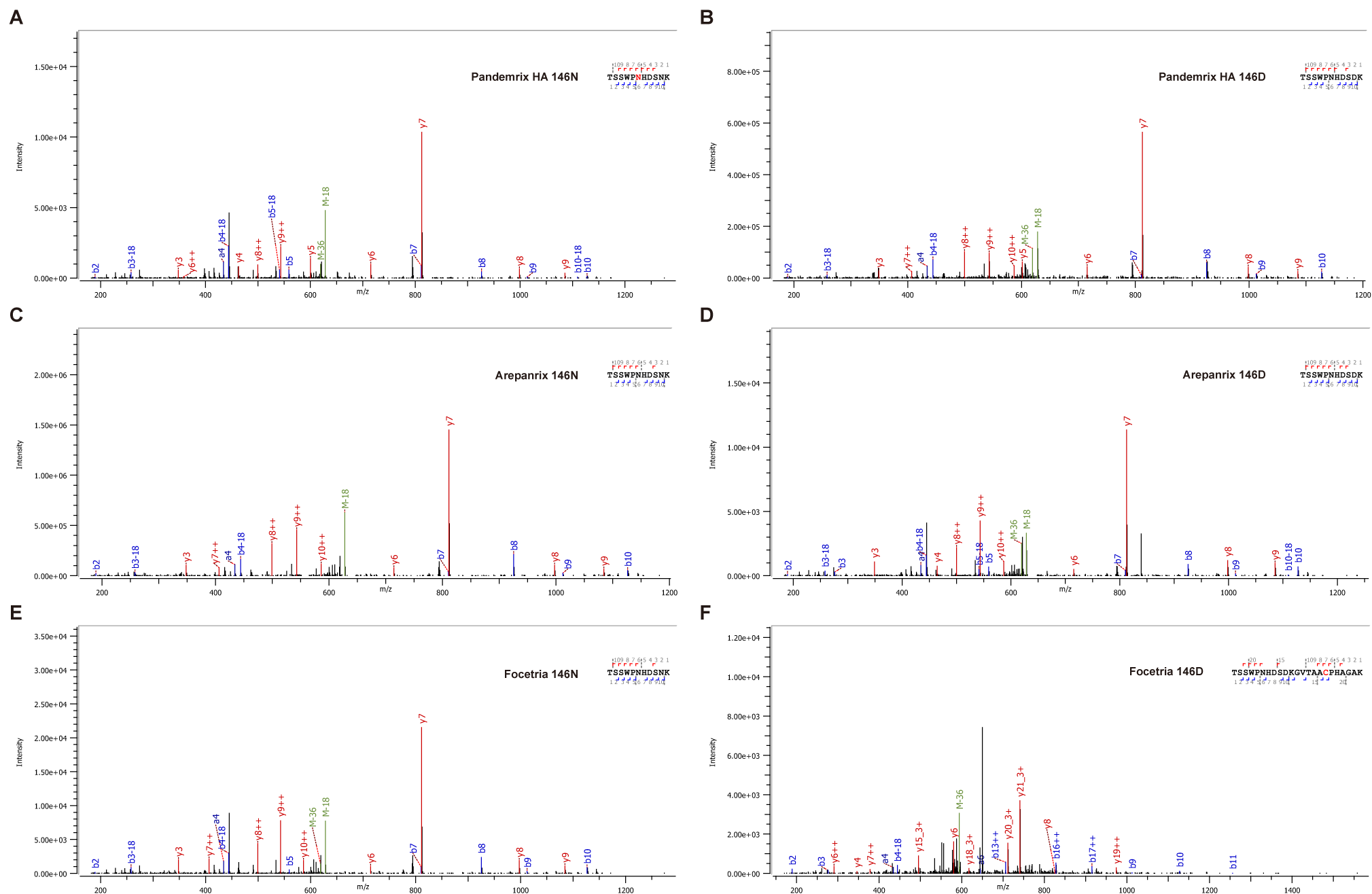

Supplement: S5 Fig — Series a/b and y of peptides are numbered from N-terminal and C-terminal ends, respectively. Associated series number denoted size of fragments in amino acid residue number, from 1 to 10. HA 146N to 146D mutation corresponded to a one Da shift. (TIF) [file pone.0187305.s007.tif]

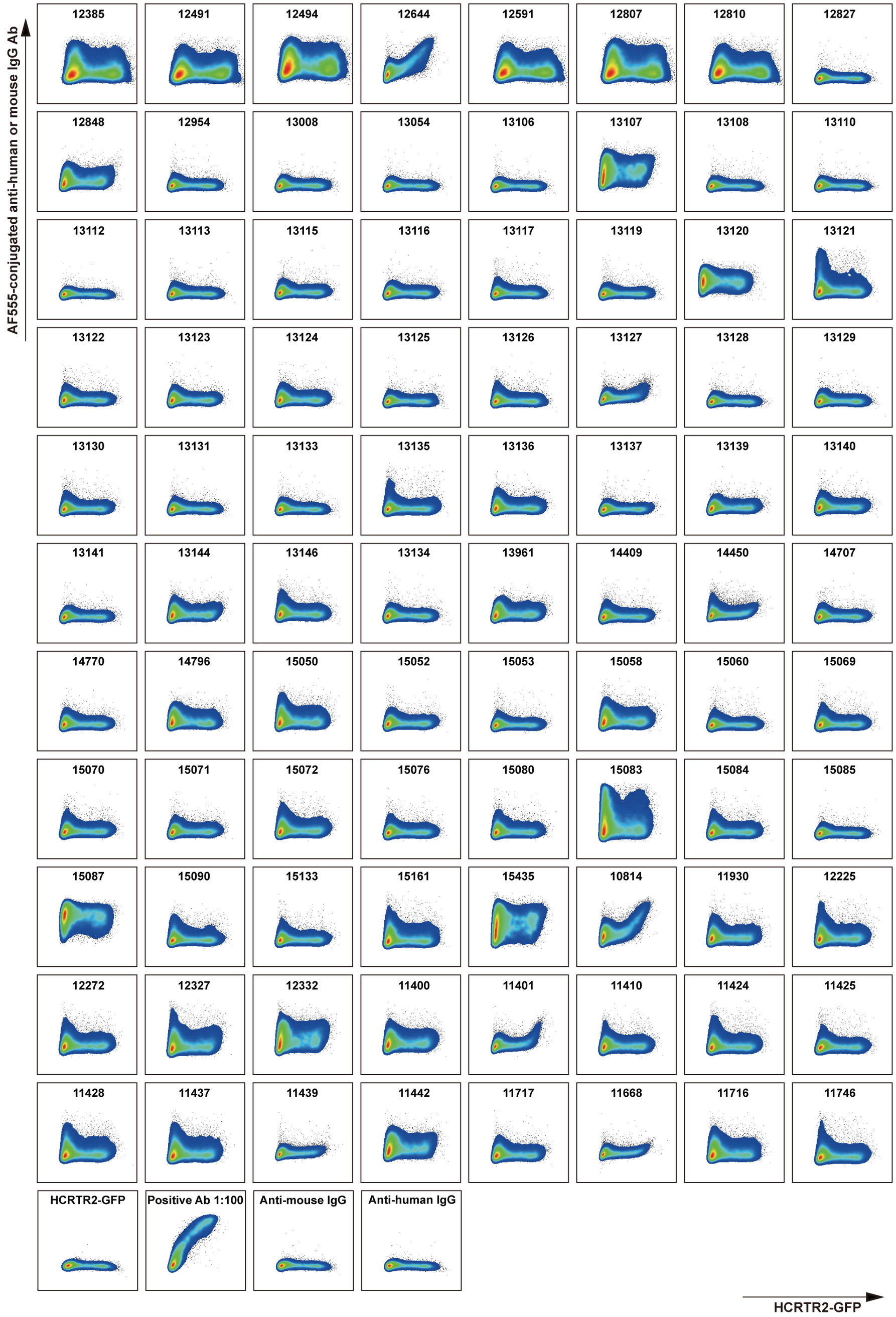

Supplement: S6 Fig — HEK293T cells with transient expression of HCRTR2-GFP were stained with positive anti-HCRTR2 antibodies (Ab) (1:100) or human sera (1:20), followed by staining with Alexa Fluor® 555 (AF555)-conjugated anti-mouse IgG or anti-human IgG (1:100), respectively. Dot plots of live single cells are shown with GFP channel (X axis) and AF555 channel (Y axis) for each sample with database identity (DbID). HEK293T cells stained with only AF555-conjugated anti-mouse IgG (Anti-mouse IgG) or anti-human IgG (Anti-human IgG) (1:100), or without any antibody staining (HCRTR2-GFP) are shown as background control. (TIF) [file pone.0187305.s008.tif]

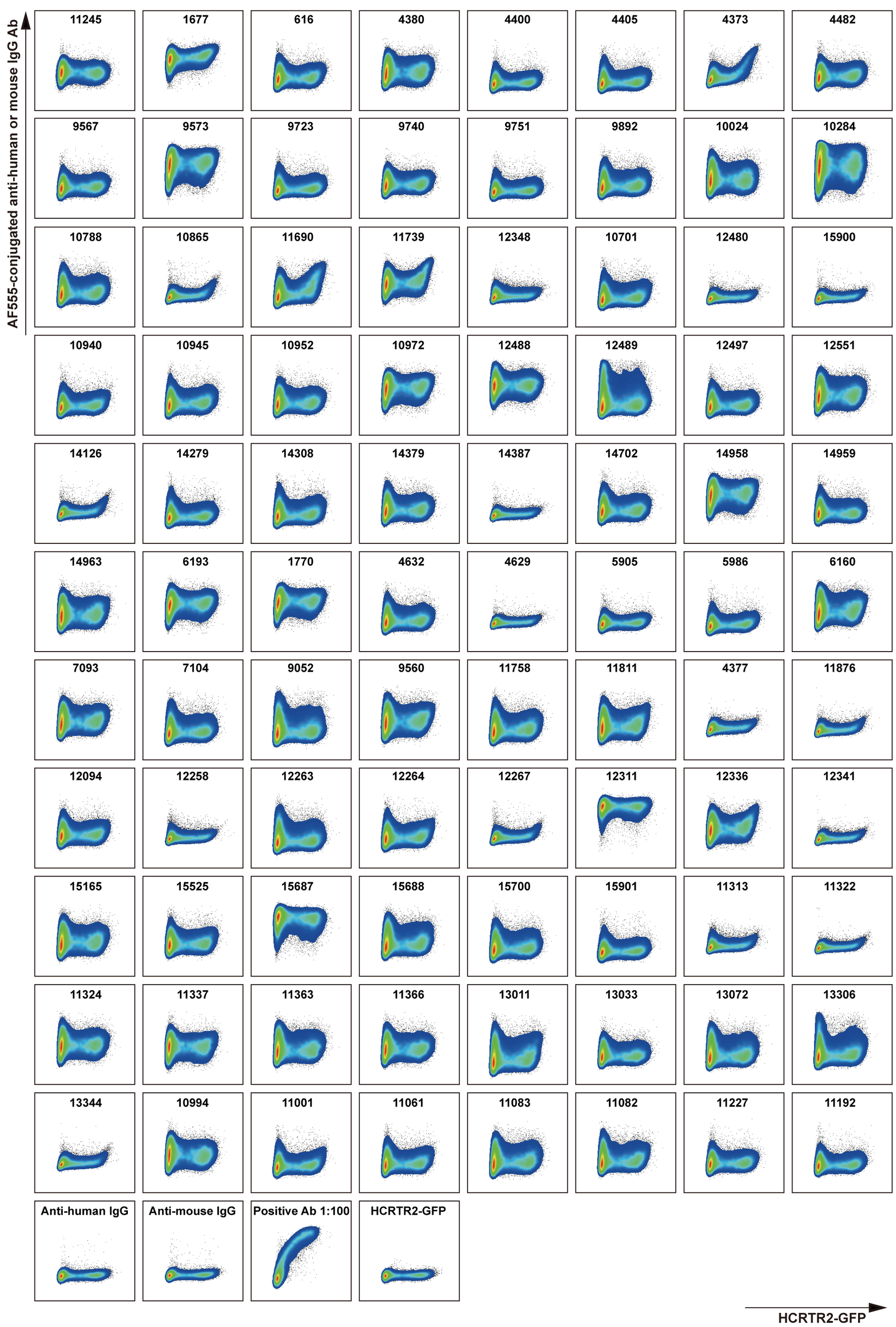

Supplement: S7 Fig — HEK293T cells with transient expression of HCRTR2-GFP were stained with positive anti-HCRTR2 antibodies (Ab) (1:100) or human sera (1:20), followed by staining with Alexa Fluor® 555 (AF555)-conjugated anti-mouse IgG or anti-human IgG (1:100), respectively. Dot plots of live single cells are shown with GFP channel (X axis) and AF555 channel (Y axis) for each sample with database identity (DbID). HEK293T cells stained with only AF555-conjugated anti-mouse IgG (Anti-mouse IgG) or anti-human IgG (Anti-human IgG) (1:100), or without any antibody staining (HCRTR2-GFP) are shown as background control. (TIF) [file pone.0187305.s009.tif]

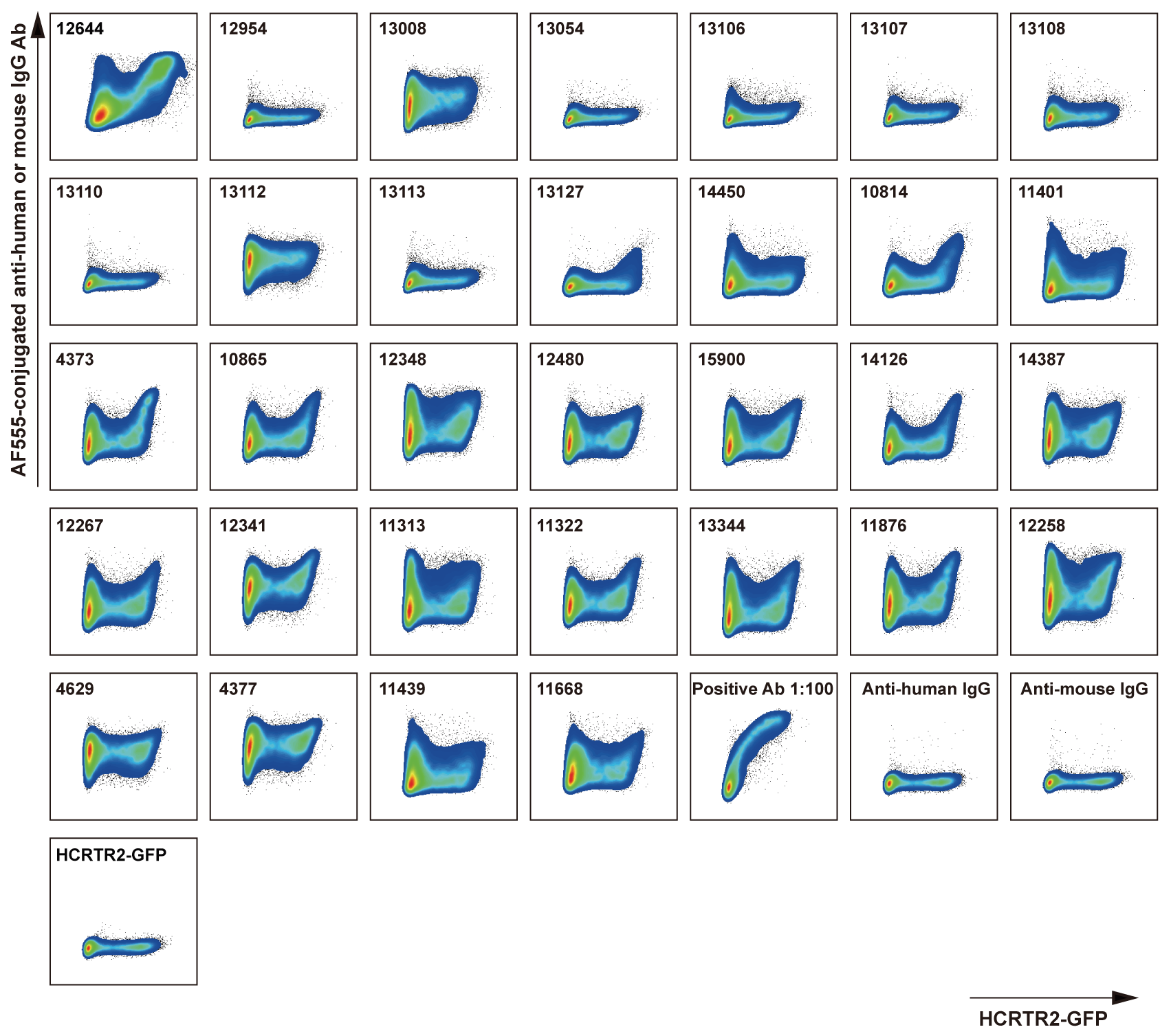

Supplement: S8 Fig — HEK293T cells with transient expression of HCRTR2-GFP were stained with positive anti-HCRTR2 antibodies (Ab) (1:100) or human sera (1:20), including potential positive sera according to results of the first screening, followed by staining with Alexa Fluor® 555 (AF555)-conjugated anti-mouse IgG or anti-human IgG (1:100), respectively. Dot plots of live single cells are shown with GFP channel (X axis) and AF555 channel (Y axis) for each sample with database identity (DbID). HEK293T cells stained with only AF555-conjugated anti-mouse IgG (Anti-mouse IgG) or anti-human IgG (Anti-human IgG) (1:100), or without any antibody staining (HCRTR2-GFP) are shown as background control. (TIF) [file pone.0187305.s010.tif]

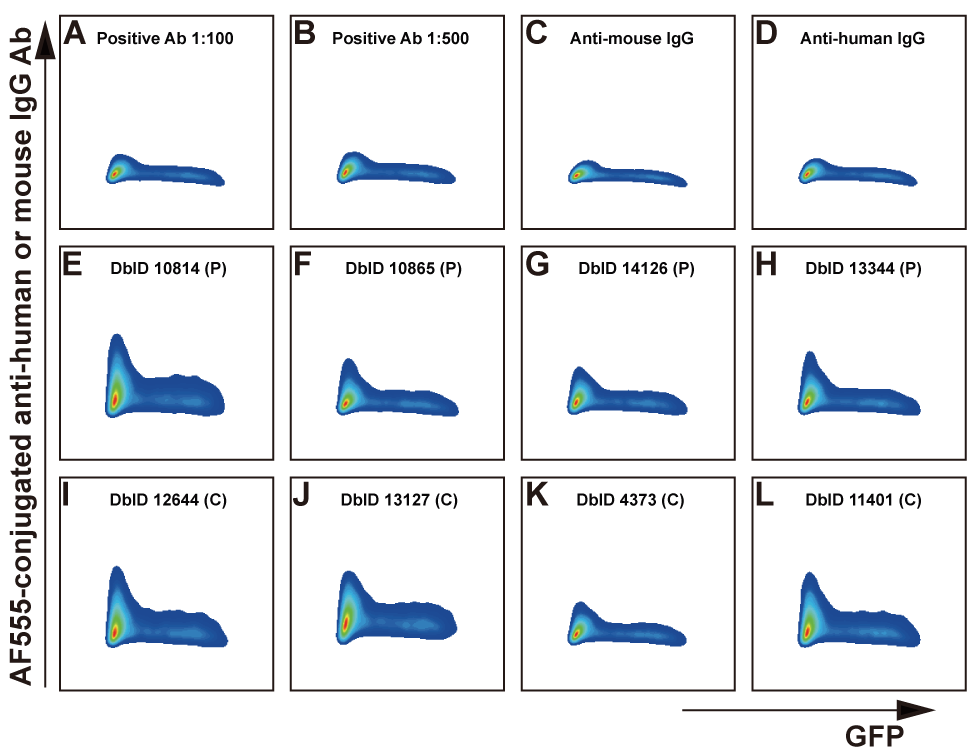

Supplement: S9 Fig — HEK293T cells with transient expression of GFP alone were stained with positive anti-HCRTR2 antibody at different dilution ratios (A and B) or human serum (1:20) (E-L), followed by staining with Alexa Fluor® 555 (AF555)-conjugated anti-mouse IgG or anti-human IgG (1:100), respectively. Dot plots of live single cells are shown with GFP channel (X axis) and AF555 channel (Y axis) for each sample with database identity (DbID). HEK293T cells stained with only AF555-conjugated anti-mouse IgG (C) or anti-human IgG (D) (1:100) are shown as background control. (TIF) [file pone.0187305.s011.tif]

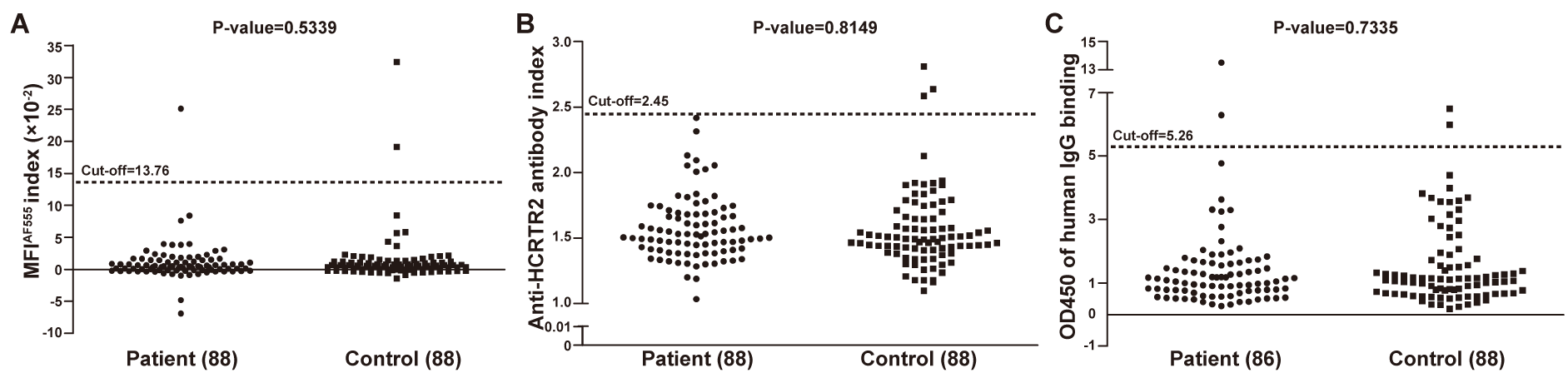

Supplement: S10 Fig — Narcolepsy patients and controls were tested using flow cytometry (A), [35S]-radiolabelled HCRTR2 binding assay (B), and in-cell ELISA using CHO-HCRTR2 (C). Each dot corresponds to one patient or a control subject. The dotted line denotes the cut-off value, the mean ± 3× SD of all healthy control subjects for each method. Values above this threshold were considered positive for anti-HCRTR2 autoantibody reaction. The numbers of each group is given. P-values are shown between patients and controls. (TIF) [file pone.0187305.s012.tif]

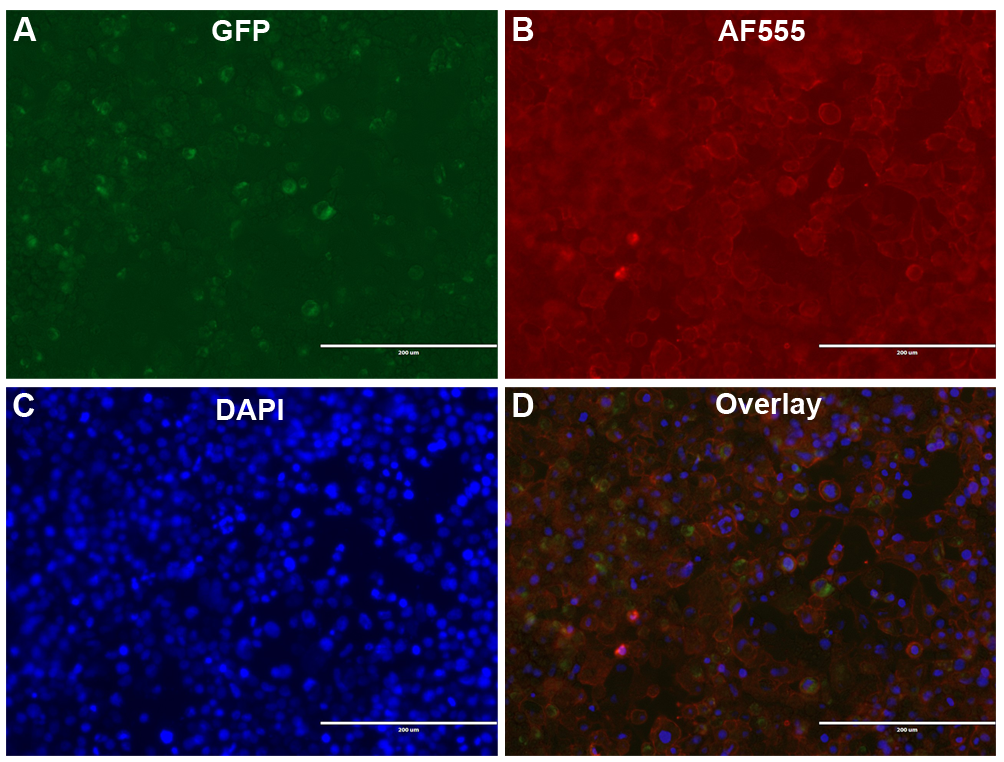

Supplement: S11 Fig — HEK293T cells transiently expressing HCRTR2-GFP were stained with positive anti-HCRTR2 antibody (1:100), followed by secondary Alexa Fluor® 555 (AF555)-conjugated antibody (1:1000), GFP channel (A), AF555 channel (B), DAPI channel (C), and overlay (D) are shown. Bar = 200 μm. (TIF) [file pone.0187305.s013.tif]
