# Supplementary figures and images for: Correction: Absence of anti-hypocretin receptor 2 autoantibodies in post pandemrix narcolepsy cases
Source: PLoS One. 2019 Mar 28;14(3):e0214340. doi: 10.1371/journal.pone.0214340 (PMC6438471; doi:10.1371/journal.pone.0214340)

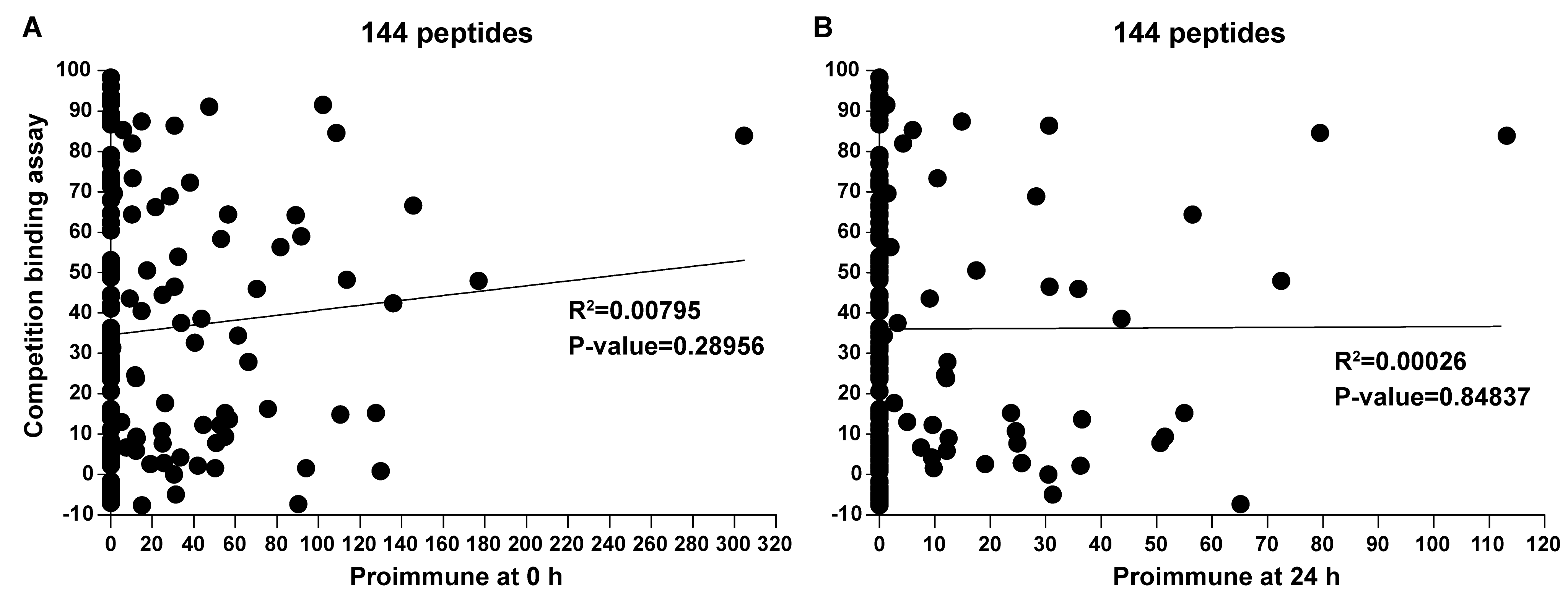

Supplement: S12 Fig — 144 peptides were tested using both assays. Note that the Bio-EBV competition results were inverted (100%-displacement) so that higher value indicate higher binding for easier comparison with the Proimmune assay. (TIF) [file pone.0214340.s001.tif]

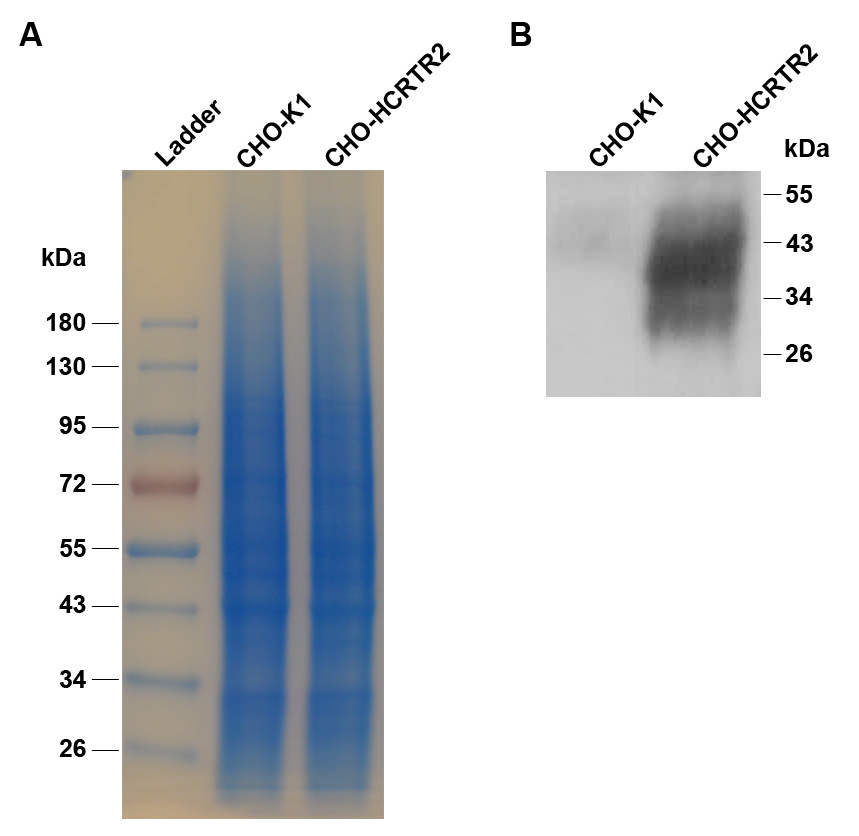

Supplement: S13 Fig — A. Coomassie blue staining of whole cell lysates used in S4 Fig of Luo et al. [1]. Note equal amount of protein in both lanes. B. Repeat western blot of the same protein lysates stained with monoclonal anti-HCRTR2 antibody (Cat# WH0003062M1-100UG, Sigma), with focus on the area of HCRTR2 molecular weight size. (TIF) [file pone.0214340.s002.tif]

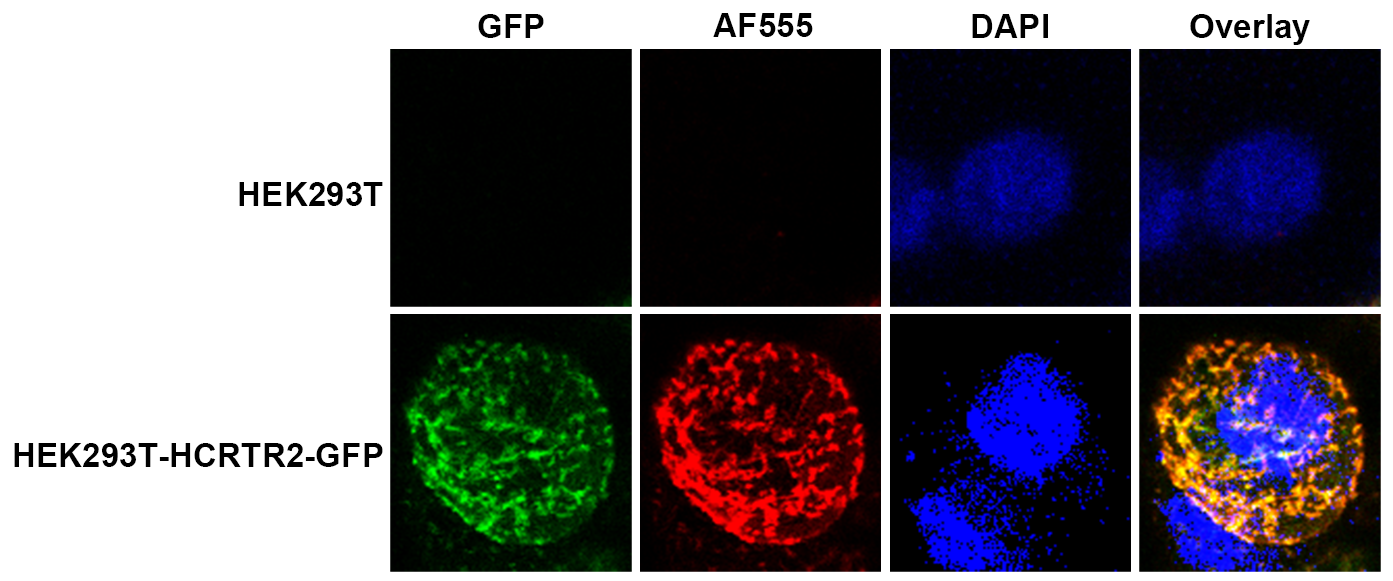

Supplement: S14 Fig — Cells were cultured and stained as described in [1] (see “Anti-HCRTR2 autoantibody detection with flow cytometry”). Images were taken using Leica TCS SP8 confocal microscope. Contrast and brightness of the digital image of only DAPI channel from HEK293T-HCRTR2-GFP were slightly adjusted for easier viewing. AF555, Alexa Fluor 555. (TIF) [file pone.0214340.s003.tif]

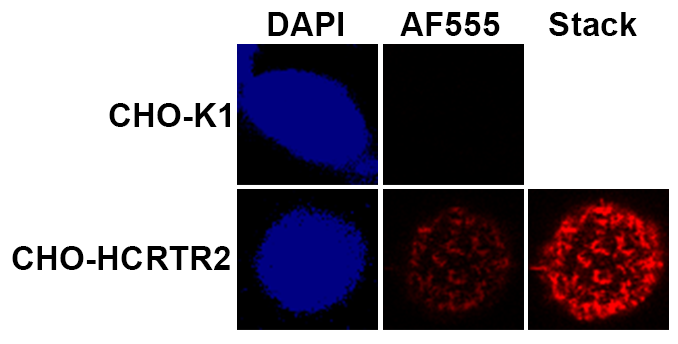

Supplement: S15 Fig — Cells were cultured and stained as described in [1] (see “Anti-HCRTR2 autoantibody detection using in-cell ELISA”). Images were taken using Leica TCS SP8 confocal microscope. These results complement our in-cell ELISA results obtained with this cell line. AF555, Alexa Fluor 555. (TIF) [file pone.0214340.s004.tif]

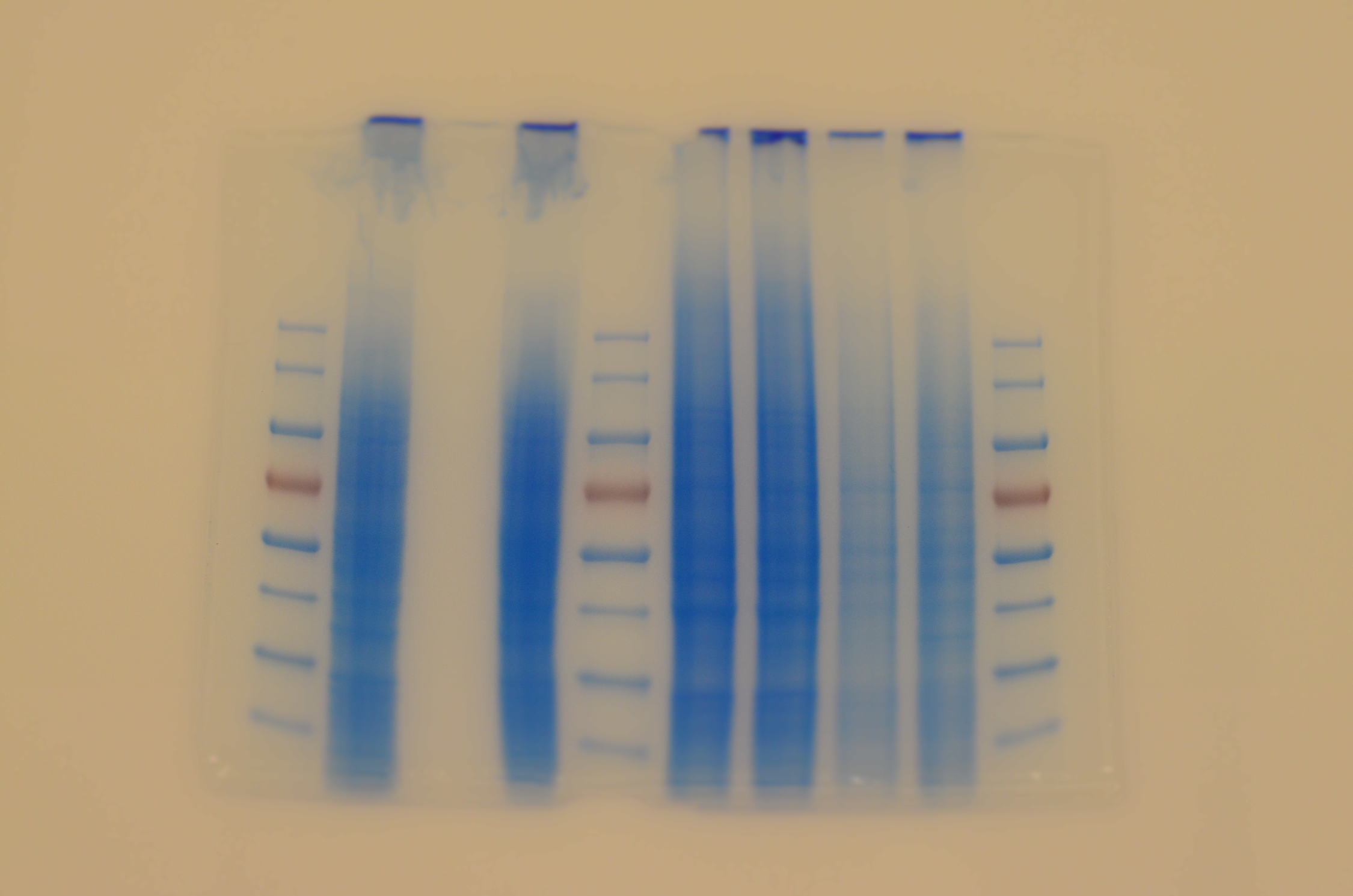

Supplement: S3 File — (TIF) [file pone.0214340.s007.tif]

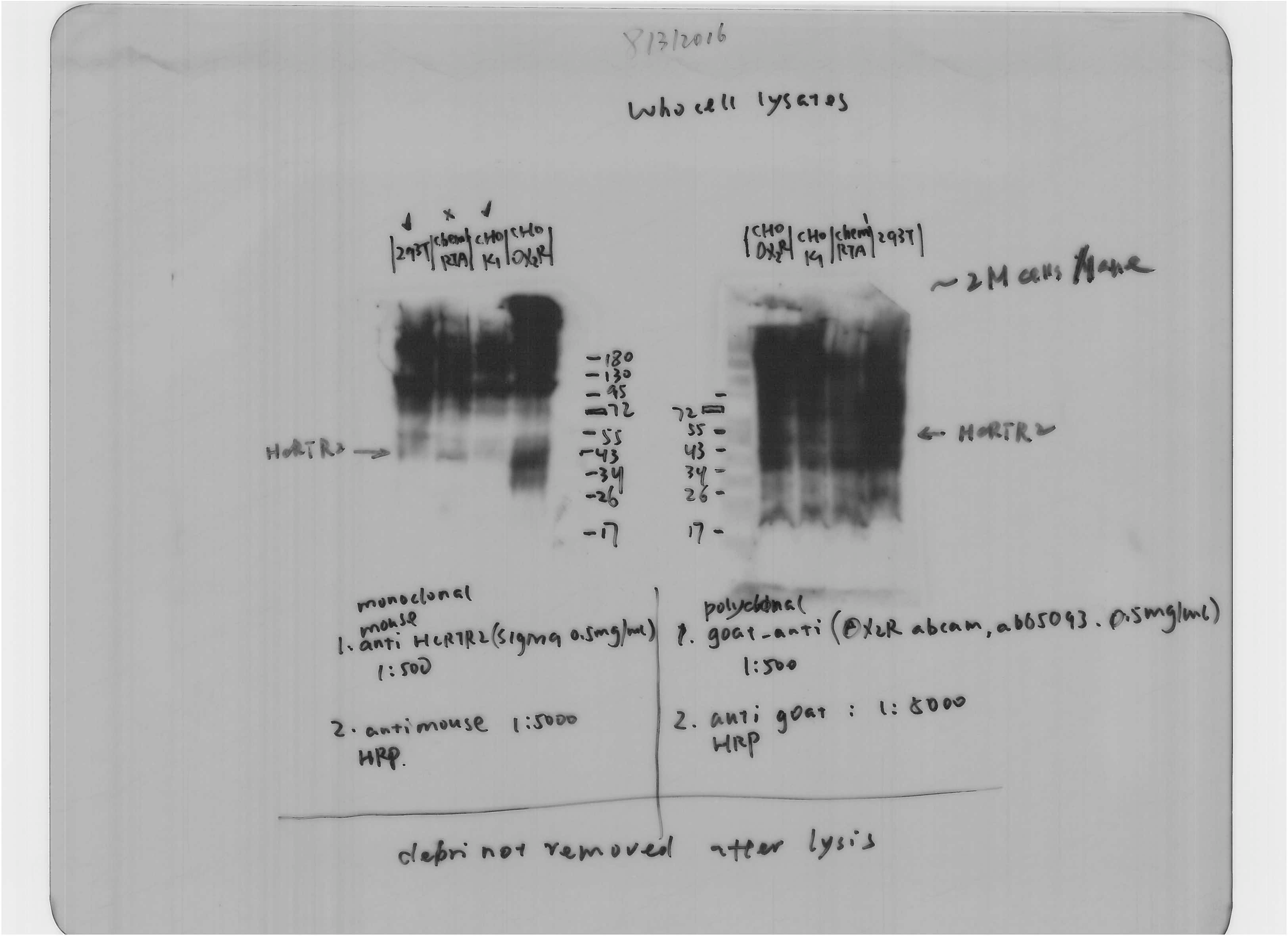

Supplement: S4 File — Note that the blot on the left, probed with the monoclonal antibody, was used in preparing S13 Fig. (TIF) [file pone.0214340.s008.tif]
